# Identification and Functional Characterization of *Squamosa* Promoter Binding Protein-Like Gene *TaSPL16* in Wheat (*Triticum aestivum* L.)

**DOI:** 10.3389/fpls.2019.00212

**Published:** 2019-02-22

**Authors:** Rufei Cao, Lijian Guo, Meng Ma, Wenjing Zhang, Xiangli Liu, Huixian Zhao

**Affiliations:** ^1^College of Life Sciences, Northwest A&F University, Yangling, China; ^2^State Key Laboratory of Crop Stress Biology for Arid Areas, College of Agronmy, Northwest A&F University, Yangling, China

**Keywords:** *Triticum aestivum* L., *TaSPL16*, identification, expression profile, functional characterization

## Abstract

Wheat (*Triticum aestivum* L.) is one of the most important crops in the world. *Squamosa* promoter binding protein-like (SPL) proteins are plant-specific transcript factors and play critical roles in plant growth and development. The functions of many *SPL* gene family members were well characterized in *Arabidopsis* and rice, in contrast, research on wheat *SPL* genes is lagging behind. In this study, we cloned and characterized *TaSPL16*, an orthologous gene of rice *OsSPL16*, in wheat. Three *TaSPL16* homoeologs are located on the short arms of chromosome 7A, 7B, and 7D, and share more than 96% sequence identity with each other. All the *TaSPL16* homoeologs have three exons and two introns, with a miR156 binding site in their last exons. They encode putative proteins of 407, 409, and 414 amino acid residues, respectively. Subcellular localization showed TaSPL16 distribution in the cell nucleus, and transcription activity of TaSPL16 was validated in yeast. Analysis of the spatiotemporal expression profile showed that *TaSPL16* is highly expressed in young developing panicles, lowly expressed in developing seeds and almost undetectable in vegetative tissues. Ectopic expression of *TaSPL16* in *Arabidopsis* causes a delay in the emergence of vegetative leaves (3–4 days late), promotes early flowering (5–7 days early), increases organ size, and affects yield-related traits. These results demonstrated the regulatory roles of *TaSPL16* in plant growth and development as well as seed yield. Our findings enrich the existing knowledge on *SPL* genes in wheat and provide valuable information for further investigating the effects of *TaSPL16* on plant architecture and yield-related traits of wheat.

## Introduction

*Squamosa* promoter binding protein-like (SPL) proteins are a diverse plant-specific transcription factor family, which are characterized by their *Squamosa* promoter-binding (SBP) domain containing a highly conserved region of 76 amino acid residues and consisting of a bipartite nuclear localization signal (NLS) and a zinc finger motif with two Zn^2+^-binding sites: Cys-Cys-His-Cys and Cys-Cys-Cys-His (Yamasaki et al., 2004; Birkenbihl et al., 2005; Yamasaki et al., 2008). They can bind specifically to the cis-element TNCGTACAA in the promoter region of their target genes (Cardon et al., 1997; Cardon et al., 1999; Birkenbihl et al., 2005). The first two *SPL* genes, *SBP1* and *SBP2*, were found in *Antirrhinum majus* and proved to be involved in the control of early flower development (Klein et al., 1996). Ever since, *SPL* genes have been identified in various plant species, such as *Arabidopsis*, maize, tomato, rice, and wheat (Cardon et al., 1999; Manning et al., 2006; Xie et al., 2006; Hultquist and Dorweiler, 2008; Zhang et al., 2014). Based on sequence analysis, 17 non-redundant *SPL* genes in *Arabidopsis* genome and 19 in rice genome were predicted (Cardon et al., 1999; Xie et al., 2006; Yang et al., 2008). These *SPL* genes vary greatly in their size and gene structure, and different *SPL* genes contain different exon number, whereas the SBP domains of all these SPLs are encoded by the first and second exons (Guo et al., 2008; Yang et al., 2008). These *SPL* genes can be separated into two types: with and without microRNA (miRNA) miR156 and 157 binding site. The *SPLs* containing miR156/157 binding site are greatly regulated by the miRNAs (Xie et al., 2006; Gandikota et al., 2007; Wang et al., 2009; Xing et al., 2010; Preston and Hileman, 2013). These *SPLs* are also divided into five different groups based on their conserved SBP domains (Zhang et al., 2014).

Functional analyses of *SPL* genes in various plant species have uncovered their important roles in plant growth and development, including leaf development (Wu and Poethig, 2006; Shikata et al., 2009), juvenile-to-adult transition (Schwarz et al., 2008), plant architecture (Stone et al., 2005; Wang et al., 2005; Jiao et al., 2010; Miura et al., 2010), vegetative-to-reproductive phase change and flowering (Gandikota et al., 2007), inflorescence branching (Jiao et al., 2010; Miura et al., 2010), organ size (Wang et al., 2008, 2012; Si et al., 2016), fruit development (Manning et al., 2006), and grain yield and quality (Wang et al., 2012; Si et al., 2016). In *Arabidopsis*, the functions of many *SPL* gene family members have been well characterized (Cardon et al., 1997; Stone et al., 2005; Wu and Poethig, 2006; Gandikota et al., 2007; Schwarz et al., 2008). In maize, *SPL* gene *teosinte glume architecture* (*tga1*) play a critical role in maize kernel domestication (Wang et al., 2005), and *SPL* gene *tasselsheath4* (*tsh4*) functions in initiation and maintenance of inflorescence branch meristem (Chuck et al., 2010). In rice, *OsSPL13* can increase grain length and yield by positive regulation of cell size in the grain hull (Si et al., 2016). *OsSPL14* boosts shoot and panicle branching and enhances grain productivity (Jiao et al., 2010; Miura et al., 2010). *OsSPL16* controls grain size, shape, and quality (Wang et al., 2012). These studies have indicated that *SPL* genes play crucial roles in regulation of plant development and yield-related traits in cereal crops.

Allohexaploid bread wheat (*Triticum aestivum* L., 2*n* = 6*x* = 42, AABBDD), which has most complex genomes with overall size of more than 16 Gb (Zimin et al., 2017; International Wheat Genome Sequencing Consortium [IWGSC], 2018), is one of the most important cereal crops for human diets worldwide. The constant increase in the global population makes high and stable yield as always a major target of wheat breeding. However, the study on functional identification of *SPL* genes in bread wheat is far behind, compared with that in rice and maize. By using bioinformatics approaches, 19 putative wheat *SPL* genes were predicted, and 10 of them were isolated from wheat cultivar Yanzhan 4110 (Zhang et al., 2014). Further study revealed that two paralogous *SPL* genes, *TaSPL20* and *TaSPL21*, were highly expressed in the lemma and palea. Over-expression of *TaSPL20*/*21* in rice had similar functions in promoting panicle branching but showed distinctive functions during seed development. *TaSPL20* over-expression resulted in enlarged seed size and increased 1000-grain weight (TGW) while *TaSPL21* over-expression did not affect seed size but significantly reduced TGW (Zhang et al., 2017). Based on sequence analysis, 58 *SPL* genes were predicted in the chromosome-based draft sequence of bread wheat cultivar Chinese Spring (CS; International Wheat Genome Sequencing Consortium [IWGSC], 2014), and ectopic expression of *TaSPL3* and *TaSPL6* in *Arabidopsis* revealed their functions in regulating flowering time and promoting biomass accumulation (Wang et al., 2015). A previous study reported that rice *OsSPL16* is a positive regulator of cell proliferation, and higher expression of *OsSPL16* promotes cell division and grain filling, leading to increases in grain width and yield in rice near-isogenic lines (NILs). Contrarily, *OsSPL16* loss-of-function associates with the formation of a more slender grain and better quality of appearance (Wang et al., 2012). Accordingly, we hypothesized that the homologous gene of rice *OsSPL16* in bread wheat should have functions in regulation of wheat grain size and yield. But to date, little is known about the homolog of *OsSPL16* in bread wheat and its function.

The major objective of this study is to explore the biological functions of the homolog of *OsSPL16* in bread wheat. We cloned *TaSPL16* in wheat and analyzed its phylogenetic relationship with *SPL*s from other plant species. Moreover, we analyzed the expression pattern of *TaSPL16* and explored its preliminary biological functions in plant growth and development by ectopic expression of *TaSPL16* in *Arabidopsis*. The findings in this study will provide valuable information for further uncovering the important roles of *TaSPL16* in high yield wheat breeding.

## Materials and Methods

### Plant Materials and Growth Conditions

Winter wheat (*T. aestivum* L.) cultivar “Shaan 512” with large grain weight (about 54 g/thousand grain weight) was used in the experiments for *TaSPL16* cDNA and genomic DNA (gDNA) cloning. Wheat plants were grown in a greenhouse with a light period of 16/8 h day/night (regulated with supplementary light), and day/night temperature regime of 20–25/15–18°C, and watered as needed. Besides, the plants were exposed to a temperature of 4°C for 40 days to achieve complete vernalization at two-leaf stage. The samples of root, leaf, flag leaf, developing panicles in different length (1–2, 3–4, 5–6, and 7–8 cm), and developing seeds at 5, 10, 15, 20 days post anthesis (DPA) were collected, three independent biological replicates for individual tissues/organs being included.

Wheat cv. CS nullisomic-tetrasomic (NT) lines, a series of lines with each missing one pair of chromosomes that is replaced by an extra pair of homoeologous chromosomes, were used for chromosome localization of *TaSPL16* in bread wheat. Seeds of these NT lines were germinated and young seedlings were grown in plant incubator. The leaf samples were collected at three-leaf stage.

The seeds of *Arabidopsis* (ecotype Columbia-0) were sterilized and sowed after 2 days imbibition at 4°C. Plants were grown at 22°C under 16/8 h light/dark cycle in phytotron, with relative humidity of 70% and light intensity of 130–150 μmol/m^2^/s.

### Nucleic Acid Isolation

Genomic DNA was extracted from the young leaf samples of wheat or *Arabidopsis* according to the cetyltrimethylammonium bromide (CTAB) method (Porebski et al., 1997). Total RNA of different tissues was isolated using the TRIZOL reagent (Invitrogen) method according to the manufacturer’s protocol, and digested with RNA-free DNase I (Invitrogen) to remove DNA contamination. The quality of the DNA and the RNA samples isolated were assessed by 0.8 and 2.0% agarose gel electrophoresis, respectively.

### cDNA Cloning and Sequence Analysis of *TaSPL16* in Wheat

For cDNA cloning, the first-strand cDNA was synthesized using total RNA isolated from 3–4 and 5–6 cm young panicles. To clone cDNA highly homologous to *OsSPL16* in wheat, *OsSPL16* cDNA sequence (GenBank accession number: AK109469; Wang et al., 2012) was used as a “query” to blast against the wheat EST database^1^, and an EST (GenBank accession number: CK207354) with 1161 bp in length covering only 27% of the *OsSPL16* cDNA was obtained. Because of the wheat EST not long enough to cover the whole CDS of the *OsSPL16* cDNA sequence and the shortage of information on wheat genome sequence and gene models when we conducted this experiment in 2013, the EST was further used to BLAST against the NCBI nucleotide database^2^, and a full-length cDNA (GenBank accession number: AK374598) from barley with high identity (83%) was obtained. Therefore, the full-length cDNA of barely was used to design a primer pair cTaSPL16-F/R to clone cDNA of *TaSPL16* in wheat by reverse transcriptase-polymerase chain reaction (RT-PCR), the sequence of the primer pair being shown in Supplementary Table S1. Because the 5′-region of the putative SPL16 sequence is highly GC-rich, 5% DMSO was added into PCR reaction to efficiently amplify the target sequence. The PCR reaction was 50 μL volume including 100 ng cDNA, plus 300 pmol of each primer, 0.2 mM of each dNTP, 1.5 mM MgCl_2_, 5% DMSO, and 1 U of *LA Taq* polymerase (TakaRa, Dalian, China). The PCR program was as follows: 5 min at 94°C; then 35 cycles of 30 s at 95°C, 30 s at 58°C, and 1 min 20 s at 72°C; plus a final extension step of 10 min at 72°C. The PCR products were recovered and cloned into the pMD19-T vector (TakaRa, Dalian, China), and 20 clones randomly chosen were sequenced.

### Cloning and Sequence Analysis of Genomic DNA of *TaSPL16*

Allohexaploid wheat was expected to have three homoeologous genes of *TaSPL16*, each on A, B, and D subgenomes. In order to design primers to amplify gDNA of the *TaSPL16* homoeologs in wheat, cDNA sequence of *TaSPL16* cloned was used as a query to BLAST against wheat whole genome shot-gun database^3^, two contigs from *Aegilops tauschii* (Genbank accession number: AOCO010294339) and *Triticum urartu* (Genbank accession number: AOTI010433783) highly homologous were identified, with 9650 and 6661 bp in length, respectively. Alignments of *TaSPL16* cDNA sequence with the two contigs found that the contig from *A. tauschii* (DD genome) covers the whole CDS of *TaSPL16* and upstream 2650 bp region, whereas the contig from *T. urartu* (AA genome) only contains partial coding region and its downstream of *TaSPL16.* The predicted *TaSPL16* homoeologous sequences were presumably 5000 bp long, and the 5’-regions were highly GC-rich (∼78%). Therefore, the gDNA of *TaSPL16* were separated into four subsection and four primer pairs g1F/R to g4F/R were designed based on an alignment of the above two contigs to amplify the whole genomic sequences of *TaSPL16* (Supplementary Figure S1 and Supplementary Table S1). For the fragments that could not be amplified with the above primer pairs, genome-specific primers were designed based on genome-walking strategy (Leoni et al., 2008). The gDNA from wheat leaves was used as a template, and the PCR reaction described above was performed to amplify the target fragments. The products were cloned into the pMD19-T vector (TakaRa, Dalian, China), and 10 more clones of each PCR product were randomly selected for sequencing. Finally, all three full-length gDNA of *TaSPL16* were assembled using subsection from each subgenome.

### Chromosome Mapping of *TaSPL16* in Hexaploid Wheat

In order to map *TaSPL16* into wheat chromosomes, the gDNA sequences of *TaSPL16* homoeologs were used as queries to BLAST against the chromosome-based draft genome sequence of hexaploid bread wheat (CS) (International Wheat Genome Sequencing Consortium [IWGSC], 2014) and a latest released fully annotated reference genome (International Wheat Genome Sequencing Consortium [IWGSC], 2018). Chromosome localization of *TaSPL16* homoeologs was further confirmed by PCR using the gDNA of the “CS” NT lines described above as templates and gene-specific primer pairs TaSPL16-A-F/R, TaSPL16-B-F/R, and TaSPL16-D-F/R (Supplementary Table S1).

### Phylogenetic Analysis

The nucleotide or amino acid sequences of *Arabidopsis SPL* genes were obtained from TAIR^4^, rice *SPL* genes were downloaded from RGAP^5^, *SPL* genes of maize and other plant species were acquired from NCBI and Phytozome v10.3^6^. Multiple alignments of the deduced amino acid sequences of *SPL* genes were performed using the CLUSTALW algorithm (Thompson et al., 1994). A phylogenetic tree was constructed by MEGA 5.05 software using neighbor-joining method with 1000 bootstrap replicates (Saitou and Nei, 1987; Kumar et al., 2008).

### Subcellular Localization of TaSPL16

To investigate the subcellular localization of TaSPL16, the CaMV35S promoter, *GFP*/fragment, and *NOS* terminator were successively cloned into plasmid *pUC18* (BioDee^®^, Beijing BioDee) to construct the transient expression vector. The coding region of *TaSPL16-7B* was amplified using the primer pair with restriction sites (TaSPL16-F:5′-CGGGATCCATGGACTGGGATCTCAAGATGC-3′ and TaSPL16-R: 5′-CCCAAGCTTCTGCCACGGGAAGGGCAGAGAT-3′, *Bam*H I and *Hind* III site underlined, respectively) and subcloned into the above transient expression vector between the CaMV35S promoter and the *GFP* gene, generating a fusion protein gene *TaSPL16-7B-GFP* driven by the CaMV35S promoter. The resulted construct p35S::*TaSPL16-7B-GFP* was confirmed by restriction analysis and sequencing. The recombinant vector was then bombarded into onion epidermal cells via a gene gun system (Bio-Rad, United States), with p35S::*GFP* as a control, and the transformed cells were incubated on 1/2 Murashige and Skoog (MS) medium (Murashige and Skoog, 1962) in light or darkness for 36–48 h at 28°C. The subcellular localization of GFP and fusion proteins TaSPL16-7B-GFP was visualized with a fluorescence microscope (Olympus, Japan).

### Transcription Activity Analysis of TaSPL16

To detect the transcription activity of TaSPL16, the coding region of *TaSPL16-7B* was fused to yeast expression plasmid pGBKT7 (Clontech) to obtain pGBKT7*-TaSPL16-7B*. The primer pair TaSPL16-F: 5′-GATCATATGGACTGGGATCTCAAGATGC-3′ (*Nde* I site underlined) and TaSPL16-R: 5′-GCGGATCCCTACTGCCACGGGAAGGGCAGA-3′ (*Bam*H I site underlined) were used to amplify the full-length coding sequence (CDS) of *TaSPL16-7B*. The recombinant construct was confirmed by restriction analysis and sequencing, and then transformed into yeast strain *Y2Hgold* (Clontech) using PEG-LiCl method (Gietz and Schiestl, 2007), with the empty pGBKT7 as the negative control. The resulted colonies were screened on the SD/-Trp and SD/-Trp/-Ade/-His yeast medium.

### Construction of *TaSPL16* Overexpression Vector and *Arabidopsis* Transformation

miR156, a most conserved miRNA family in plant kingdom, can target many members of *SPL* gene family and regulate plant development by controlling expression levels of *SPL* genes (Wu and Poethig, 2006; Yu et al., 2015). In order to generate *SPL* overexpression *Arabidopsis* plants, preventing the cleavage of the target mRNA by endogenous miR156 in transgenic plants is the first factor to be considered when constructing overexpression vector of the target gene, which can be achieved by mutating miR156-targeted site in *SPL* (Wang et al., 2009). Therefore, a mutant of *TaSPL16-7B* gene at its miR156 target site (*mTaSPL16-7B*) was generated according to the protocol of site overlap extension-PCR-mutagenesis (Ho et al., 1989), using mutant forward primer (mTaSPL16-F: 5′-CTGATTGTGCTCTCTCACTACTATCTTCCT-3′) and reverse primer (mTaSPL16-R: 5′-AGGAAGATAGTAGTGAGAGAGCACAATCAG-3′) with ORF of *TaSPL16-7B* cDNA as a template. The ORF of *TaSPL16-7B* and *mTaSPL16-7B* was amplified using primer pairs TaSPL16-*Bam*H I -F/TaSPL16-FLAG-R and TaSPL16-*Bam*H I -F/FLAG-*Spe* I -R, respectively, and used to construct expression vectors under the control of the maize ubiquitin promoter (pUBI) with pTCK303 (Biovector *NTCC Inc*., Beijing) as a backbone. The primer sequences are as follows: TaSPL16-*Bam*H I -F: 5′-CGGGATCCATGGACTGGGATCTCAAGATGC-3′, TaSPL16-FLAG-R: 5′-CTATTTGTCGTCGTCGTCCTTGTAGTCCTGCCACGGGAAGGGCAGA-3′, and FLAG-*Spe* I -R: 5′-GACTAGTCTATTTGTCGTCGTCGTCCTTGTAGTC-3′. The resulted constructs pUBI*::TaSPL16-7B* (called as pUBI*::TaSPL16* for short) and pUBI*::mTaSPL16-7B* (called as pUBI::*mTaSPL16* for short) were confirmed by sequencing, and then transformed into *Arabidopsis* wild-type (Col), respectively, by *Agrobacterium tumefaciens*-mediated floral-dip method (Zhang et al., 2006). Transgenic plants were screened on MS medium with 25 mg/L Hygromycin, and independent transgenic homozygous lines were obtained by self-breeding and screening with Hygromycin. To assess the effects of *TaSPL16* on transgenic plants, wild-type and the obtained homozygous transgenic lines were grown at green houses as described above.

### Quantitative Real-Time Reverse Transcriptase-Polymerase Chain Reaction

Quantitative real-time reverse transcriptase-polymerase chain reaction (qRT-PCR) was conducted to determine the expression levels of *TaSPL16* gene across different wheat tissues/organs. Because three *TaSPL16* homoeologous genes have very high sequence identity, it is difficult to detect the expression level of each homoeolog. Therefore, we designed a homoeolog-specific primer pairs and detected the total mRNA abundance of three *TaSPL16* homoeologs. The *TaSPL16* cDNA products of individual wheat tissues were normalized using wheat *GAPDH* (GenBank accession number: EF592180) as an internal reference gene, which was confirmed to be relatively stable in the tested wheat tissues in our previous study (Ma et al., 2015). Three biological replicates were included, and triplicates were performed for technical replicates. All the primers used for qRT-PCR are listed in Supplementary Table S1.

The expression levels of target genes in *Arabidopsis* were also quantified by qRT-PCR. The cDNA products of wild-type plants and transgenic lines were normalized using *Tubulin beta 2* (AT5G62690) as an internal reference gene. Three biological replicates were included. The qRT-PCR was performed in triplicate for each RNA sample/primer combination. The primer sequences used for qRT-PCR are shown in Supplementary Table S1. The program of qRT-PCR was as follows: denaturation at 95°C for 30 s, followed by 40 cycles of 95°C for 5 s and 60°C for 30 s. The qRT-PCR was operated on iCycler iQTM Multi-Color Real Time PCR Detection System (Bio-Rad, Hercules, CA, United States) using SYBR Green Master Mix (TakaRa, Dalian, China). For each PCR, the specificity of the amplification was validated and the threshold cycle above background was calculated using Bio-Rad iCycler software, and PCR efficiency close to 100%.

The relative expression levels of the target genes were calculated by an improved ΔΔ method (Pfaffl et al., 2002). Error bars in all figures showing qRT-PCR data represented the standard deviation that was calculated from the original CT (cycle threshold) values; and *P*-values were estimated using hypothesis test (Student’s *t*-test).

### Phenotype Measurement and Statistical Analysis

Flowering time of *Arabidopsis* plant was accounted by scoring bolting days (from sowing time to the primary inflorescence reaching a height of 0.5 cm), 10 more plants were measured for each of the independent transgenic lines and wild type. For measurement of *Arabidopsis* root length, at least ten 7-day-old seedlings were detected for each of the independent transgenic lines. For measurement of silique length, ten well-developed siliques from the middle of the primary inflorescence of each plant were detected, at least 10 plants for individual transgenic lines. For measurement of *Arabidopsis* seed size, dried seeds from the middle of the primary inflorescence of wild-type plants and individual transgenic lines were photographed on black cloth using a Olympus camera and quantified by Image J software^7^. Then the photographs were converted to binary images, the seed size was measured using the “Analyze particles” function of Image J and was represented by the projective areas of a seed. At least 50 seeds were measured for each plant, and at least 10 plants for individual transgenic lines. To determine seed weight and biomass per plant, total seeds per plant and the aboveground parts of the whole plant were weighed when the plants became maturity, using a Sartorius ME5-0CE microbalance, 10 more plants were measured for individual transgenic lines. Moreover, silique number per plant was counted and plant height was measured, 10 more plants for each of transgenic lines.

Statistical analysis was performed using SPSS 19.0 software (SPSS Inc., 2010).

## Results

### Cloning and Characterization of *TaSPL16* in Wheat

The cDNA sequence highly homologous to *HvSPL16* and *OsSPL16* in wheat was amplified by RT-PCR, cloned into pMD19-T vector and 20 clones were randomly chosen to sequence. The results showed that all the cloned cDNA sequences were 1299 bp in length with high identity (>99%). These fragments contained the whole coding region of TaSPL16 protein based on alignments with the full-length cDNA of barely, and encoded a putative protein of 409 amino acid residues (Supplementary Figure S2). The deduced protein contain a SBP domain consisting of a highly conserved region of 76 amino acid residues that included a bipartite NLS and a zinc finger motif with two Zn^2+^-binding sites: Cys-Cys-His-Cys and Cys-Cys-Cys-His (Supplementary Figure S2), these are typical characteristics of SPL protein family (Yamasaki et al., 2004, 2008). Moreover, there is a miR156 binding site that consists of 21 nucleotides in the cDNA sequence (Supplementary Figure S2).

**FIGURE 1:**
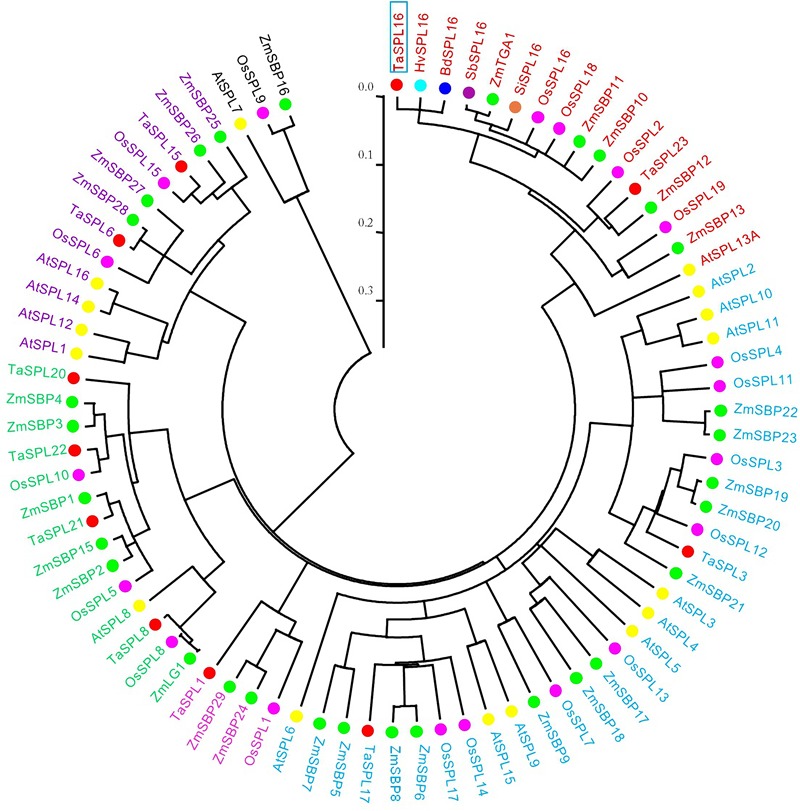
Phylogenetic analysis of *TaSPL16* and other *SPL* genes based on the conserved SBP domain. The tree was constructed by alignment of a total of 79 *SPLs*, including 16 from *Arabidopsis*, 19 from rice, 30 from maize, 11 from wheat, and the orthorolog of *SbSPL16* from sorghum, barley, millet, *Brachpodium distachyon*, using ClustalW, and MEGA5.05 was used to generate a neighbor-joining tree with 1000 bootstrap replicates. The scale bar indicates the average number of amino acid substitutions per site. *TaSPL16* is marked with blue box. Filled circles in different colors represent different species; genes in the same clades are with the same color. At, *Arabidopsis*; Bd, *Brachypodium distachyon*; Hv, *Hordeum vulgar*; Sb, *Sorghum bicolor*; Si, *Setaria italica*; Os, *Oryza sativa*; Ta, *Triticum aestivum*; Zm, *Zea mays*.

Many *SPL* genes have been isolated from various plant species, such as rice and wheat (Xie et al., 2006; Zhang et al., 2014). In order to evaluate the phylogenetic relationship of the *TaSPL16* cloned above with those *SPLs* isolated from wheat and other species, a total of 79 *SPL*s were used for phylogenetic analysis based on the SBP domain, including 16 from *Arabidopsis* (Yang et al., 2008), 19 from rice (Xie et al., 2006; Yang et al., 2008), 30 from maize (Hultquist and Dorweiler, 2008), 10 from wheat (Zhang et al., 2014), the *TaSPL16* isolated above, and those *SPL16* from other cereal crops such as sorghum, barley, millet, and *Brachpodium distachyon.* The result showed that all the 79 *SPLs* were clustered into six groups, and each group included at least one member from *Arabidopsis*, rice, and maize (Figure 1), suggesting that the main characteristics of *SPL* gene family were established before the split of *Arabidopsis*, rice, maize, and wheat. The orthologous *SPL16* genes (*TaSPL16, HvSPL16*, and *BdSPL16*) from wheat, barley, and *B. distachyon*, the paralogous genes *OsSPL16*/*OsSPL18* in rice, and the orthologous genes *TaSPL23* and *OsSPL2* from wheat and rice, together with *AtSPL13A* in *Arabidopsis* were clustered in the same group (Figure 1). In order to understand the evolutionary relationships among *TaSPL16, TaSPL23, OsSPL16*/*OsSPL18*, and *AtSPL13A*, we further conducted phylogenetic analysis using *TaSPL16, TaSPL23*, and all members of *SPL* gene family in *Arabidopsis* and rice. The result also showed *TaSPL16, OsSPL16*/*OsSPL18, TaSPL23*, and *OsSPL2* together with *AtSPL13A* belong to the same *SPL* gene lineage (Figure 1 and Supplementary Figure S3). Accordingly, we concluded that the full-length cDNA of the *SPL* we obtained was the ortholog of *HvSPL16* and *OsSPL16.* The sequence was temporarily designated as *TaSPL16-cDNA1*.

Wheat is allohexaploid, and it is expected to have three *TaSPL16* homoeologs, each from A, B, and D subgenomes. The strategy of subsection cloning was used to isolate gDNA of *TaSPL16* in wheat by PCR, due to too long putative sequences. All four primer pairs gF1/gR1 to gF4/gR4 designed for amplifying each subsection of each *TaSPL16* homoeolog from A, B, or D subgenome could produce PCR products with expected-size, except for primer pair gF2/gR2 (Supplementary Figure S1 and Supplementary Table S2). Twenty independent clones of the PCR products generated with each of the three primer pairs were randomly chosen for sequencing. Finally, two different sequences (at least three clones for each type) were obtained for each primer pair, and the resulted six fragments were separately mapped in A, B, or D subgenome based on alignments of these sequences with the two contigs (Genbank accession AOCO010294339 and AOTI010433783) from *A. tauschii* (DD genome) and *T. urartu* (AA genome), respectively (Supplementary Table S2). For cloning the remaining fragment of *TaSPL16* in A subgenome of wheat, a subgenome-specific primer pair gF2/gAR2 were designed based on alignment of its two adjacent fragments sequenced with the two contigs mentioned above. Similarly, for the two missing subsections in B subgenome, two pairs of subgenome-specific primer gF2/gBR2 and gBF4/gBR4 were designed according to alignment of their adjacent sequenced fragments with the two contigs and used to clone the target fragments. Whereas, for the three missing subsections in D subgenome, three pairs of genome-specific primer (gDF1/gDR1, gF2/gDR2, and gDF3/gDR3) for *TaSPL16* homoeolog in D subgenome were designed based on alignment of the corresponding sequences in A and B subgenomes with the contig from D genome (Supplementary Figure S1 and Supplementary Table S2). Finally, all the target subsection were cloned and sequenced, and the gDNA sequences of the *TaSPL16* homoeologs in A, B, and D subgenomes were assembled, with 5061, 4824, and 5229 bp in length (Supplementary Table S2), and temporarily designated as *gTaSPL16-A, gTaSPL16-B*, and *gTaSPL16-D*, respectively. The three *TaSPL16* homoeologs have high identity (≥96%), only with some single nucleotide polymorphisms and some insertions/deletions mainly in their intron sequences, and they all contained whole length of *TaSPL16* gDNA including coding and non-coding region (Supplementary Figure S4 and Supplementary Table S3).

We used the gDNA sequences of the *TaSPL16* homoeologs as queries to BLAST against the chromosome-based draft sequence of bread wheat (CS) genome (International Wheat Genome Sequencing Consortium [IWGSC], 2014) and the latest released fully annotated reference genome of bread wheat (International Wheat Genome Sequencing Consortium [IWGSC], 2018). The results showed that *gTaSPL16-A, gTaSPL16-B*, and *gTaSPL16-D* are located on short arms of wheat chromosome 7A, 7B, and 7D, respectively (Supplementary Tables S4, S5). Transcripts for the three *TaSPL16* homoeologs were also identified in the annotated reference genome (Supplementary Table S5). A previous study reported that 58 putative *SPL* genes were predicted in the Chromosome Survey Sequence of hexaploid wheat CS by searching gene models and scaffolds as well as the CS genome contigs (International Wheat Genome Sequencing Consortium [IWGSC], 2014), however, many of the hits were only partially aligned with the complete SBP domain that was used as a query, and further experiment work is needed to confirm these bioinformatics data in hexaploid wheat (Wang et al., 2015). In the previous study, two gene models (Traes_5AL_637B13721.1 and Traes_5BL_96FDFC152.1) on 5AL and 5BL were predicted for *TaSPL16* (Wang et al., 2015). This is not consistent with our result above. To clarify this, we further conducted a PCR-test by using CS NT lines and genome-specific primer pairs, and the result exhibited that *TaSPL16-A, TaSPL16-B*, and *TaSPL16-D* isolated in our study located on wheat chromosome 7A, 7B, and 7D rather than 5A, 5B, and 5D (Supplementary Figure S5). Thus, *TaSPL16-A, TaSPL16-B*, and *TaSPL16-D* were named as *TaSPL16-7A, TaSPL16-7B*, and *TaSPL16-7D*, respectively.

**FIGURE 2:**
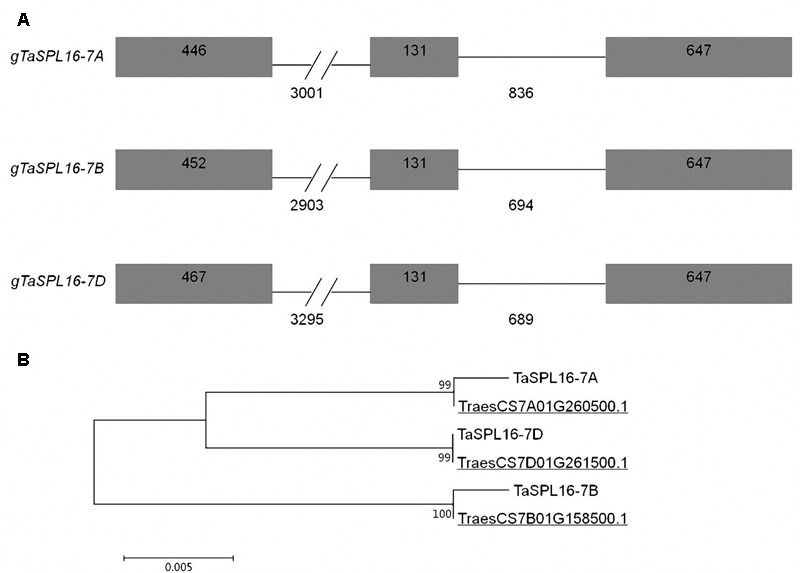
The structures of *TaSPL16* homoeologs and alignment of the deduced TaSPL16 proteins in wheat. **(A)** The structures of the *TaSPL16* homoeologs. Black boxes indicate exons, and the lines between exons denote introns. The longer introns are condensed as signified by a double slash (//). Numbers indicate exon or intron size (bp). **(B)** ClustalW alignment of the deduced protein TaSPL16-7B with TaSPL16-7A and TaSPL16-7D. The underlines indicate the protein ID of TaSPL16 in the fully annotated reference genome of wheat (International Wheat Genome Sequencing Consortium [IWGSC], 2018). Scale bar indicates the average number of amino acid substitutions per site. The values next the branches indicate the identity of the two corresponding TaSPL16 protein.

Characterization of the three *TaSPL16* homoeologs, *TaSPL16-7A, TaSPL16-7B*, and *TaSPL16-7D*, showed that they all include three exons and two introns, and the first intron of each *TaSPL16* homoeolog is about 3000 bp in length, much longer than the exons and the second intron (Figure 2A). At the last exon of each *TaSPL16* homoeolog, there is a miR156 binding site that consists of 21 nucleotides (Figure 2A and Supplementary Figure S4), suggesting that *TaSPL16* homoeologs might be regulated by miR156. The putative CDS of *TaSPL16-7A, TaSPL16-7B*, and *TaSPL16-7D* is 1224, 1230, and 1245 bp in length, respectively, and encodes putative protein of 407, 409, and 414 amino acid residues, respectively (Figure 2A and Supplementary Figure S6). The protein TraesCS7A01G260500.1, TraesCS7B01G158500.1, and TraesCS7D01G261500.1 that correspond to the deduced protein TaSPL16-7A, TaSPL16-7B, and TaSPL16-7D were also found in the latest fully annotated reference genome of wheat cultivar CS (International Wheat Genome Sequencing Consortium [IWGSC], 2018). TaSPL16-7A and the corresponding TraesCS7A01G260500.1, TaSPL16-7B and TraesCS7B01G158500.1, TaSPL16-7D and TraesCS7D01G261500.1 have more than 99% sequence identity (Figure 2B), suggesting that the *TaSPL16* homoeologs isolated in the present study and their deduced proteins are correct. Furthermore, TaSPL16-7A, TaSPL16-7B, and TaSPL16-7D displayed high sequence identity (93.5–95.4%) (Figure 2B, Supplementary Table S3, and Supplementary Figure S6). All of the three deduced TaSPL16 proteins contained a conserved SBP domain, with the typical zinc-binding sites Cys-Cys-His-Cys and Cys-Cys-Cys-His (Supplementary Figure S6). In fact, the *TaSPL16-cDNA1* cloned above was the transcript of *TaSPL16-7B.*

We further characterized the features of those *SPL16* genes and the other homologous genes that belong to the same cluster as *TaSPL16* in the phylogenetic tree shown in Figure 1. The result showed that these *SPL* genes have the same structural characteristics, with three exons and two introns, their SBP domain being encoded by the first and the second exon, and a miR156 binding site that is located on the last exon (Supplementary Figure S7A). Furthermore, alignment of SPL16 proteins from cereal crops (including barley, rice, sorghum, maize, and millet) with AtSPL13A from *Arabidopsis* showed that SPL16 proteins from the cereal crops have high sequence identity, with 88% between TaSPL16 and HvSPL16, 60% between TaSPL16 and OsSPL16. While TaSPL16 and AtSPL13A showed high identity (78%) in their SBP domain but low identity (33%) in whole protein sequence (Supplementary Figure S7B). These reflect divergence of the same *SPL* gene lineage in evolution of the plant SPL gene family.

### Subcellular Localization and Transcription Activity of TaSPL16-7B Protein

**FIGURE 3:**
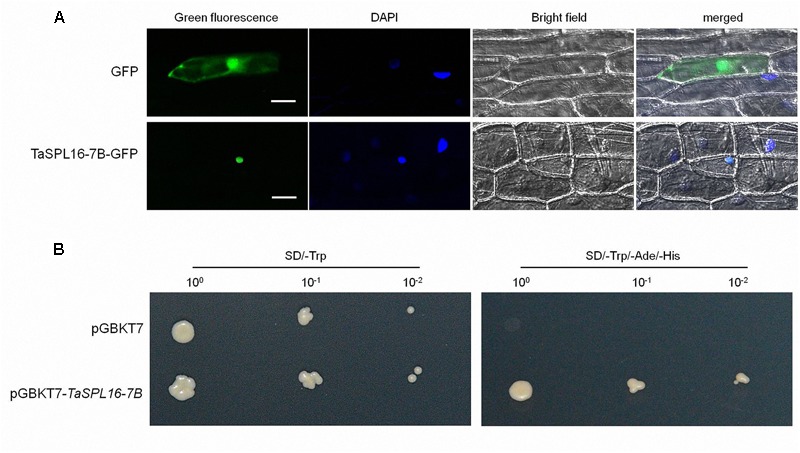
Subcellular location and transcription activity analysis of TaSPL16-7B protein. **(A)** Subcellular location of TaSPL16-7B. Transient expression vector p35S::*TaSPL16-7B-GFP* and control vector p35S::*GFP* were introduced into onion epidermal cells. GFP and fusion protein TaSPL16-7B-GFP, and nucleus region were monitored by laser scanning confocal microscopy. DAPI, 4’, 6-diamidino-2-phenylindole, a blue-fluorescent DNA stain that used for indicating nucleus region; Bright field used as control for cell integrity; merges are the overlap of GFP and DAPI. Scale bars: 50 μm. **(B)** Transcription activity analysis of TaSPL16-7B protein. The yeast colonies with three different dilution 10^-1^, 10^-2^, 10^-3^ were plated on the screening medium SD/-Trp and SD/-Trp/-Ade/-His, respectively. The empty pGBKT7 was used as a negative control. SD, Synthetic Dropout Medium; SD/-Trp, Trp-defective SD; SD/-Trp/-Ade/-His, Trp-, Ade-, and His-defective SD.

As a transcription factor, SPL protein should be located in the nucleus. In order to confirm the subcellular localization of TaSPL16, we developed a *TaSPL16-7B-*containing transient expression vector p35S::*TaSPL16-7B-GFP* to express TaSPL16-7B-GFP fusion protein in onion epidermal cells, with p35S::*GFP* as a control. The result was as expected, GFP was located in both cytoplasm and the nucleus, but TaSPL16-7B-GFP was localized only in the cell nucleus (Figure 3A).

Transcription activity analysis of TaSPL16 was validated in yeast using TaSPL16-7B. The recombinant construct of the pGBKT7*-TaSPL16-7B* was transformed into the yeast *Y2Hgold* and screened on the yeast medium SD/-Trp and SD/-Trp/-Ade/-His. As expected, yeast colonies with pGBKT7*-TaSPL16* can grow on the SD/-Trp and SD/-Trp/-Ade/-His, while those with pGBKT7 just grow on SD/-Trp, indicating that TaSPL16 had the transcription activity (Figure 3B).

### Spatiotemporal Expression Patterns of *TaSPL16* in Wheat

**FIGURE 4:**
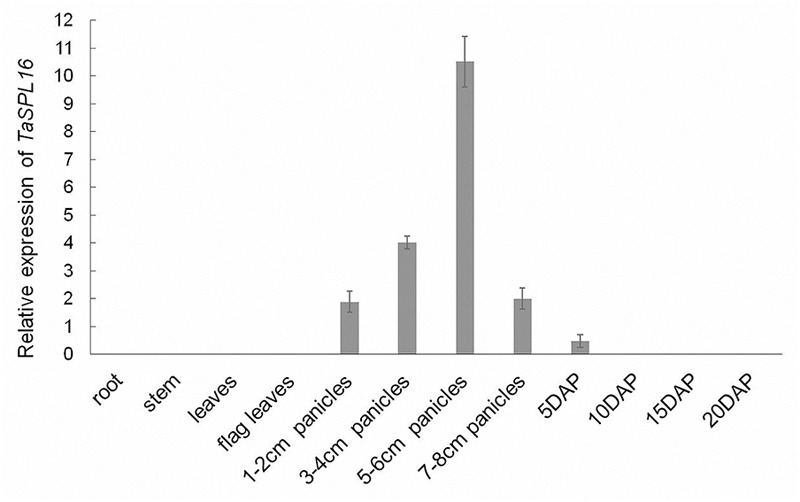
Expression profiles of *TaSPL16* in different wheat tissues. Quantitative real-time reverse transcriptase-polymerase chain reaction was conducted to detect total mRNA level of *TaSPL16* homoeologs in different tissues, *GAPDH* (GenBank accession number: EF592180) being used for normalization; DAP means days after pollination.

Knowledge about the spatiotemporal expression profile of *TaSPL16* might provide a clue that where TaSPL16 functions. So, we tested the total mRNA abundance of the three *TaSPL16* homoeologs in different wheat tissues/organs, including root and leaf of seedling, flag leaf, young panicles, and seeds at different development stages. The results showed that *TaSPL16* is highly expressed in young developing panicles, lowly expressed in developing seeds and almost undetectable in vegetative tissues (Figure 4), suggesting that *TaSPL16* might play important role in developmental panicles and seeds of wheat. Similar result was obtained in rice *OsSPL16* that was preferentially expressed in panicles of 7 cm in length and hardly accumulated in the root, culm, leaf sheath, shoot meristem, and young panicle < 1 cm in length (Wang et al., 2012).

### Ectopic Expression of *TaSPL16* in *Arabidopsis* Regulated Phase Transition

We constructed *TaSPL16* expression vector pUBI::*TaSPL16* and *mTaSPL16* expression vector pUBI::*mTaSPL16* by introducing seven silent mutations at the miRNA target site of *TaSPL16* (Supplementary Figure S8A). Sixteen and 12 independent transgenic lines were generated for pUBI::*TaSPL16* and pUBI::*mTaSPL16* transformation, respectively, and 10 more independent transgenic homozygous lines for each of different genotypes were obtained for later analysis.

We first detected the mRNA abundance of *TaSPL16* across different developmental stages in wild-type plants and transgenic lines *TaSPL16-OEs* expressing non-mutated *TaSPL16* and m*TaSPL16-OEs* expressing miR156-resistance mRNA of *TaSPL16*, respectively. The result showed that at post-germination and seedling stages, *mTaSPL16-OEs* had the highest expression level of miR156-resistance *TaSPL16*, followed by *TaSPL16-OE*s that had median level of *TaSPL16*, and wild type had none (Supplementary Figures S8B,C), indicating that *mTaSPL16-OEs* over-accumulated miR156-resistance target mRNA, compared with transgenic *TaSPL16-OEs*. While *TaSPL16-OEs* and *mTaSPL16-OEs* had similar level of *TaSPL16* mRNA, but both had remarkable higher levels than wild-type plants at reproductive stage (Supplementary Figure S8B). This is mainly attributed to the variation of miR156 abundance during plant growth and development, which is most abundance in juvenile plant but significantly declines in adult plant and reproductive organs (Wu and Poethig, 2006).

We next observed phenotypes of *TaSPL16-OEs* and *mTaSPL16-OEs* as well as wild-type plants across different developmental stages. Compared with wild type and *TaSPL16-OEs, mTaSPL16-OEs* seedlings exhibited a delay in the emergence of vegetative leaves (3–4 days late), *mTaSPL16-OE3* and *TaSPL16-OE3* being shown as representatives (Supplementary Figure S9A) because of similar phenotypes among independent *mTaSPL16-OEs* or independent *TaSPL16-OEs*. This indicates that the phenotype was caused specifically by the silent mutations and deregulation of *TaSPL16* from miR156. In addition, *mTaSPL16-OEs* also showed a significant decrease in leaf initiation rate, compared with wild-type plants and the transgenic lines *TaSPL16-OEs* (Supplementary Figures S9B,C). A similar phenomenon was also found in *mAtSPL13* over-expression transgenic lines of *Arabidopsis* (Martin et al., 2010).

**FIGURE 5:**
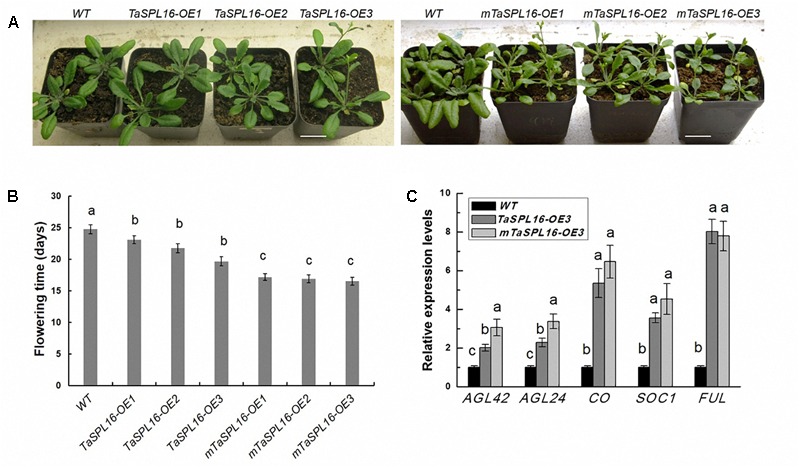
Overexpression of *TaSPL16* promotes early flowering in *Arabidopsis*. WT, wild-type *Arabidopsis*; *TaSPL16-OEs*, transgenic lines expressing non-mutated *TaSPL16-7B*; *mTaSPL16-OEs*, transgenic lines expressing miR156-resistance mRNA of *TaSPL16-7B*. **(A)** Phenotypes of wild-type and homozygous transgenic lines at three week old. Scale bar: 2 cm. **(B)** Comparison of flowering time of wild-type and transgenic lines. Flowering time of *Arabidopsis* plant is accounted by scoring bolting days (from sowing to the primary inflorescence reaching a height of 0.5 cm). **(C)** Relative expression levels of known flowering-related genes in wild-type and transgenic lines. *TaSPL16-OE3* and *mTaSPL16-OE3* are shown as representatives, because similar phenotypes were observed among independent *mTaSPL16-OEs or TaSPL16-OEs*. The value of each column represents mean ± SD (*n* = 10). Different letters at top of each column indicate a significant difference among genotypes at *P* < 0.05 determined by Tukey’s HSD test.

Furthermore, compared with wild-type plants, transgenic *mTaSPL16-OEs* and *TaSPL16-OEs* exhibited significantly early flowering (5–7 days early) (Figure 5A,B). We also determined the expression levels of several known flowering-related genes, *AGL42, AGL24, CO, SOC1*, and *FUL*, by qRT-PCR using 10-day-old seedlings of wild type and these transgenic lines. The results showed that all the transgenic lines had significantly higher mRNA levels of these flowering-related genes than wild-type plants, with *TaSPL16-OE3* and *mTaSPL16-OE3* shown as representatives (Figure 5C), this suggesting that *TaSPL16* promoted expression of SPL-targeted *MADS* box genes in *Arabidopsis*, such as *AGL42, AGL24, SOC1*, and *FUL*.

### Ectopic Expression of *TaSPL16* in *Arabidopsis* Enlarged Organ Size

**FIGURE 6:**
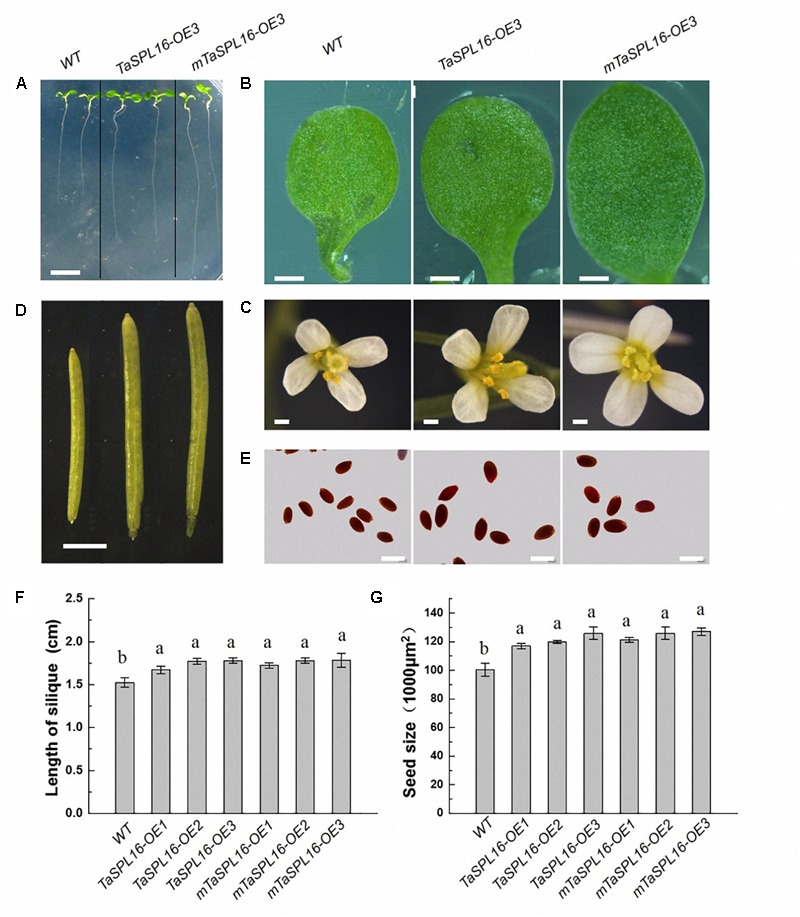
Overexpression of *TaSPL16* enlarges organs of *Arabidopsis*. WT, wild-type *Arabidopsis*; *TaSPL16-OE3*, a transgenic line expressing non-mutated *TaSPL16-7B*; *mTaSPL16-OE3*, a transgenic line expressing miR156-resistance mRNA of *mTaSPL16-7B*. *TaSPL16-OE3* and *mTaSPL16-OE3* are shown as representatives because similar phenotypes were observed among independent *TaSPL16-OE* lines or independent *mTaSPL16-OE* lines. **(A)** The roots of 7-day-old seedlings. Scale bar: 5 mm. **(B)** Cotyledon of 7-day-old seedlings. Scale bar: 0.5 mm. **(C)** Floral organs. Scale bar: 1 mm. **(D)** Siliques. Scale bar: 2 mm. **(E)** Mature seeds. Scale bar: 0.5 mm. **(F,G)** Comparison of silique length and seed size (represented by the projective areas of a seed) of wild-type and these transgenic plants. Data given as mean ± SD (*n* = 10). Different letters at top of each column indicate a significant difference among genotypes at *P* < 0.05 determined by Tukey’s HSD test.

We further investigated all organs of *Arabidopsis* wild-type plants and transgenic lines *TaSPL16-OEs* and *mTaSPL16-OEs.* Apparent differences were observed in different organs among wild-type plants and these transgenic lines, with *TaSPL16-OE3* and *mTaSPL16-OE3* shown as representatives of transgenic lines (Figure 6A–E), because of similar phenotypes among independent *TaSPL16-OEs or* independent *mTaSPL16-OEs*. At seedling stage, *TaSPL16-OEs* and *mTaSPL16-OEs* displayed longer root and larger cotyledons than wild-type plants (Figure 6A,B). In reproductive phase, all the transgenic lines exhibited enlarged floral organs, including petals, stamens, and stigmas, compared to the wild-type plants (Figure 6C). *TaSPL16-OEs* and *mTaSPL16-OEs* also produced significant longer siliques, compared to the wild-type plants (Figure 6D), with an average of 1.8–2 cm long in transgenic lines, while 1.5 cm in wild-type plants (Figure 6F). Besides, the transgenic lines produced much more seeds per silique, compared to the wild-type plants, with approximately 60–70 seeds in transgenic plants while 45–55 seeds in wild-type. The most notable thing is that the transgenic lines showed apparently larger seed size, compared to the wild-type plants (Figure 6E,G). These findings suggested that *TaSPL16* had functions in regulating organ size of *Arabidopsis*.

### Ectopic Expression of *TaSPL16* in *Arabidopsis* Affected Yield-Related Traits

To investigate the effect of *TaSPL16* on plant seed yield, we further tested yield-related traits of these transgenic lines and the wild-type plants, including plant height, silique number per plant, 100-seed weight, seed weight per plant, and harvest index (Supplementary Figures S10A–F). Compared with the wild-type plants, the transgenic lines *TaSPL16-OEs* and *mTaSPL16-OEs* exhibited greatly increased plant height and 100-seed weight (Supplementary Figures S10A,C) and significantly reduced silique number per plant (Supplementary Figure S10B). The *mTaSPL16-OE* lines had less silique number per plant than the *TaSPL16-OE* lines (Supplementary Figure S10B). However, the *TaSPL16-OE* lines and wild-type plants had similar levels in seed weight per plant, which was significantly higher than those of the *mTaSPL16-OE* lines (Supplementary Figure S10E). This result may occur because the enlarged effects of *TaSPL16* on seed size (Figure 6G) and 100-seed weight (Supplementary Figure S10C) at the whole-plant level were offset by the reduced silique number per plant that was differential between *TaSPL16-OEs* and *mTaSPL16-OEs* (Supplementary Figure S10B). It was also observed that the *TaSPL16-OE* lines and the *mTaSPL16-OE* lines have less branching, compared with wild type, *TaSPL16-OE3* and *mTaSPL16-OE3* being shown as representatives (Supplementary Figure S10G). We counted biomass per plant (*n* = 10) of these transgenic lines and wild-type plants and found that the *TaSPL16-OE* lines had the highest biomass per plant, followed by the wild-type plants, and the *mTaSPL16-OE* lines had the lowest (Supplementary Figure S10D). We further detected the harvest index (the proportion of total seed yield to total aerial biomass; *n* = 10) of these transgenic lines and wild-type plants. The result showed that these transgenic lines significantly decreased in harvest index, compared with wild-type plants (Supplementary Figure S10F), suggesting that ectopic expression of *TaSPL16* in *Arabidopsis* caused a reduction in harvest index.

In order to investigate whether the effect of *TaSPL16* overexpression on seed size was indeed direct or merely an indirect consequence of the less branching and the reduced silique-number in transgenic lines, we limited the branch and the silique number in wild-type plants and the transgenic *mTaSPL16-OE* lines, by cutting off a number of branches and siliques on the wild-type and transgenic plants and leaving only so many that their combined seed number is comparable per plant. Then we compared the seed size from these plants, and found that the seed size in either wild-type plants or *mTaSPL16-OE* lines did not increase, *mTaSPL16-OE3* being shown as a representative of *mTaSPL16-OEs* (Supplementary Figure S10H). This implied that the effect of *TaSPL16* overexpression on seed size is direct.

## Discussion

### Molecular Characterization and Phylogenetic Relationship of *TaSPL16* in Wheat

Nearly all members of *SPL* gene family were identified in *Arabidopsis*, rice, and maize (Xie et al., 2006; Hultquist and Dorweiler, 2008; Yang et al., 2008). In contrast, research on wheat *SPL* genes is lagging behind, due to the lack of availability of a high-quality wheat genome sequence previously. Until recently, members of *SPL* gene family in wheat were predicted based on sequence analysis, and some of them were isolated experimentally (Zhang et al., 2014; Wang et al., 2015). In the present study, the gDNA of *TaSPL16* homoeologs (*TaSPL16-7A, TaSPL16-7B, and TaSPL16-7D*) and the cDNA of *TaSPL16-7B* were first isolated by homology cloning technique and genome walking strategy based on the draft genome sequences of the *T. urartu* and *A. tauschii* (Jia et al., 2013; Ling et al., 2013), before the release of the chromosome-based draft sequence of bread wheat (CS) genome (International Wheat Genome Sequencing Consortium [IWGSC], 2014). The *TaSPL16* homoeologs have very high sequence identity (≥96%) with each other (Supplementary Figure S4 and Supplementary Table S3), and they all contain three exons and two introns, with a miR156 binding site in their last exon (Figure 2A and Supplementary Figure S4). Phylogenetic analysis showed that orthologous genes *TaSPL16* and *HvSPL16* in wheat and barley, *TaSPL23* and *OsSPL2* in wheat and rice, and paralogous genes *OsSPL16/OsSPL18* in rice as well as *AtSPL13A* in *Arabidopsis* belong to the same lineage of *SPL* gene family (Figure 1 and Supplementary Figure S3). A similar result was also obtained previously using ten members of *SPL* gene family from wheat and all members from *Arabidopsis* and rice (Zhang et al., 2014). These suggest that the genes of the plant *SPL* family evolved by gene duplication or divergence, and the paralogous genes *TaSPL16/TaSPL23* in wheat and *OsSPL16/OsSPL18* in rice might be the consequences of gene duplication or divergence of *AtSPL13A* after separation of wheat, rice, and *Arabidopsis*. It was proposed that an emerging pattern of sub-functionalization, neo-functionalization and possible convergent evolution following both ancient and recent gene duplication (Preston and Hileman, 2013). Accordingly, we guess that *TaSPL16* and *OsSPL16* should have similar functions, while *SPL16* genes in cereal crops and *AtSPL13A* in *Arabidopsis* might have either similar functions or sub-functionalization.

### *TaSPL16* Has Multiple Functions in Regulation of Plant Development and Yield-Related Traits

Accumulating evidences showed that *SPL* genes are pivotal and functionally diverse in regulating plant growth and development (Cardon et al., 1997; Wang et al., 2009; Miura et al., 2010; Wang et al., 2012; Preston et al., 2016). The vast majority of these results are from *Arabidopsis* and rice. Recently, the gene functions of the *SPL* gene family in wheat have been gradually uncovered. *TaSPL17/TaSPL20/TaSPL21* mainly expressed in the shoot apical meristem at seedling stage and the ear at late booting, while *TaSPL6/TaSPL15* exhibited the opposite expression pattern, this hinting that *TaSPL17/20/21* and *TaSPL6/15* might have different functions in regulating plant growth and development (Zhang et al., 2014). A further study revealed that ectopic expression of *TaSPL20* or *TaSPL21* in rice had similar functions in promoting panicle branching; however, *TaSPL20* and *TaSPL21* had different functions during seed development. Over-expression of *TaSPL20* resulted in enlarged seed size and increased TGW while over-expression of *TaSPL21* had no effect on seed size but significantly reduced TGW (Zhang et al., 2017). In the present study, we found that *TaSPL16* is preferentially expressed in young developing panicles, with low expression level in early developmental seeds and no expression in vegetative tissues (Figure 4). A similar expression pattern was also found for *OsSPL16* in rice (Wang et al., 2012). These facts further strengthen our conjecture that *TaSPL16* should have similar functions with *OsSPL16* in rice. Ectopic expression of *mTaSPL16* or *TaSPL16* caused early flowering (5–7 days earlier than wild type). More interestingly, ectopic expression of *TaSPL16* in *Arabidopsis* generated enlarged organ sizes, such as longer root, larger cotyledon, longer silique, and larger seed size (Figure 6). These phenomena were also observed in transgenic *Arabidopsis* or transgenic rice plants overexpressing *OsSPL16* (Wang et al., 2012). Unfortunately, compared with wild-type plants, the transgenic lines (both *TaSPL16-OEs* and *mTaSPL16-OEs*) exhibited less branching (Supplementary Figure S10G), resulting in a significant reduction in silique number per plant and harvest index (Supplementary Figures S10B,F), although they exhibited increased seed size and 100-seed weight (Supplementary Figures S10A,C). But the effects of *TaSPL16* on seed size were not attributed to the less branching and the reduced silique-number in these transgenic plants, because the limitation of branch and silique number per plant did not increase the seed size in either wild-type plants or the transgenic lines (Supplementary Figure S10H). It was also reported that constitutive overexpression of *OsSPL16* in rice caused fewer panicle branches and grains (Wang et al., 2012). This suggested that *TaSPL16* had similar functions as *OsSPL16* did in regulation of plant or panicle architecture. Interestingly, analyzing the effect of the alleles at *qGW8* (synonymous with *OsSPL16*) on rice yield-related traits showed that the NIL-GW8 plants with higher expression of *OsSPL16* and NIL-gw8 plants with lower expression showed no difference in plant or panicle architecture, but exhibited clearly distinct grain sizes, NIL-GW8 producing a larger grain (14.9%) and a greater TGW (Wang et al., 2012). Whether similar cases exist in wheat NILs and natural populations needs further investigation.

Moreover, compared to wild type and *TaSPL16-OEs, mTaSPL16-OEs* over-accumulated much higher level of miR156-resistant *SPL16* mRNA at seedling stage (Supplementary Figures S8B,C), resulting in a delay in the emergence of vegetative leaves (3–4 days late) and a significant decrease in leaf initiation rate (Supplementary Figures S9A–C). Similar phenotypes were also found in transgenic *Arabidopsis* over-expressing miR156-resistant *AtSPL13* (Martin et al., 2010). These indicate that *TaSPL16* and *AtSPL13* has similar function at least in regulation of post-germination transition in transgenic *Arabidopsis*, because to date there is no report about the effects of *AtSPL13* overexpressing on the whole plants beyond the post-germination stage.

## Conclusion

Three *TaSPL16* homoeologs in wheat were first experimentally isolated and characterized in the present study. Our transgenic experiment demonstrated that *TaSPL16* has multiple functions in regulation of plant growth and development as well as yield-related traits. The effects of *TaSPL16* on plant development and seed yield of *Arabidopsis* are very complex. The specific expression pattern of *TaSPL16* in wheat suggests that *TaSPL16* might play important role in developmental panicles and seeds. We plan to further identify function of *TaSPL16* in regulation of wheat development and seed production by wheat transformation. Our findings in the molecular characteristics of *TaSPL16* and its regulatory role in plant development and yield-related traits enrich the existing knowledge of *SPL* genes in wheat and also provide useful information for further investigating the effect of *TaSPL16* on plant architecture and yield-related traits of wheat.

## Author Contributions

HZ conceived and designed the experiments. RC conducted the experiments. LG, MM, WZ, and XL give helps in data analysis. RC and HZ wrote the manuscript.

## Conflict of Interest Statement

The authors declare that the research was conducted in the absence of any commercial or financial relationships that could be construed as a potential conflict of interest.

## Footnotes

1 https://blast.ncbi.nlm.nih.gov/Blast.cgi

2 https://www.ncbi.nlm.nih.gov/nuccore/

3 https://www.ncbi.nlm.nih.gov/genbank/wgs/

4 http://www.arabidopsis.org/index.jsp

5 http://rice.plantbiology.msu.edu/

6 http://phytozome.jgi.doe.gov/pz/portal.html

7 http://rsb.info.nih.gov/ij/
