# Supplementary material for: Identification and Functional Characterization of Squamosa Promoter Binding Protein-Like Gene TaSPL16 in Wheat (Triticum aestivum L.)
Source: Front Plant Sci. 2019 Feb 22;10:212. doi: 10.3389/fpls.2019.00212 (PMC6401658; doi:10.3389/fpls.2019.00212)
Supplement: Supplementary file 1 [file Data_Sheet_1.PDF]

Supplementary materials

Molecular Characterization and Functional Identification of *Squamosa* Promoter Binding Protein-Like Gene *TaSPL16* in Wheat (*Triticum aestivum* L.)

Rufei Cao[1], Lijian Guo[1], Meng Ma[1], Wenjing Zhang[1], Xiangli Liu[1], Huixian Zhao[1,2*]

Correspondence: Corresponding author: Huixian Zhao hxzhao212@nwafu.edu.cn

1. Supplementary Tables

Table S1. Sequences and positions of the primers used in this study.

Primer name	Primer sequence (5' to 3')	Primer position	Uses
cTaSPL16-F	CTGGCTTGCGCTGTACGTGTCCTCCGC	5'-UTR	cDNA cloning
cTaSPL16-R	GCCACTGAAAACTACTGCCACGGGAAG	Exon3/3'-UTR	cDNA cloning
gF1	CTGGCTTGCGCTGTACGTGTCCTC	5'-UTR	gDNA cloning
gR1	GTCCGTCCGGGTTATCATCG	Intron 1	gDNA cloning
gF2	GCCTACTGAACCTGTCCGATTT	Intron 1	gDNA cloning
gR2	TGGATGGTTCAGGTTTTCGAGT	Intron 1	gDNA cloning
gF3	CAGGTGCTTTGGGACCACTTGAC	Intron 1	gDNA cloning
gR3	CATCACATCTGGCTGCGGTTTC	Intron 1	gDNA cloning
gF4	AGCGTCTTGATGGGCACAAC	Exon2	gDNA cloning
gR4	GAAACCTACTGCCACGGGAAG	Exon/3'-UTR	gDNA cloning
gAR2	TGGATGGTTCAGGTTTTCGAGT	Intron1	gDNA cloning
gBF2	AAAGATCAAGATCACATCCACACAG	Intron1	gDNA cloning
gBR2	GGTTCCGAGTAGAGGATAAACAGTAAGA	Intron1	gDNA cloning
gBDF4	GTATTGCTTAATAAGTATATGTAGCGG	Exon2	gDNA cloning
gBDR4	GAACAGCCACTGAAAACTACTGCCAC	Exon/3'-UTR	gDNA cloning
gDF1	ATAGCTACTAGGCCTACCGGGCTGA	5'-UTR	gDNA cloning
gDR1	GCCAGTTCTTGCGGTGTGATGAGTG	Intron1	gDNA cloning
gDR2	CAGGTTCCGAGTAGAAGATATACAGTA	Intron1	gDNA cloning
gDF3	TTCAATATGGTATCAGAGCCAAGAGG	Intron1	gDNA cloning
gDR3	AATAAGCAATACCTCAAGAGTTTTCC	Intron1	gDNA cloning
TaSPL16A-F	CCGTATGCATCGATCTGATGCAGA	Exon1	Chromosome localization
TaSPL16A-R	GTTCTCCTCGGTTTTAATGATCCCTT	Intron1	Chromosome localization
TaSPL16B-F	CGTAGCTCTTCTTTTTCTTTTTTGCG	Exon1	Chromosome localization
TaSPL16B-R	GAAATCCACAGGAAAAAGCAGAACAA	Intron1	Chromosome localization
TaSPL16D-F	ATCATTCTCTTCGCTCATCCGTTG	Exon1	Chromosome localization
TaSPL16D-R	GGAAGGCGGGCAATATATTTCAACA	Intron1	Chromosome localization
qTaSPL16-F	GCTGTAGAAAGCGTCTTGATGG	Exon2	qRT-PCR
qTaSPL16-R	GAGGGAGTTGATGTGCGTAATAG	Exon3	qRT-PCR
GAPDH-F	CCTTCCGTGTTCCCACTGTTG		qRT-PCR
GAPDH-R	ATGCCCTTGAGGTTTCCCTC		qRT-PCR

AGL24-F	GAGGCTTTGGAGACAGAGTCGGTGA	qRT-PCR
AGL24-R	AGATGGAAGCCAAGCTTCAGGGAA	qRT-PCR
AGL42-F	TCATGAAACCAGCAATCACGACTCA	qRT-PCR
AGL42-R	AGCCTTTCTTTCTCGGACCTTTCC	qRT-PCR
CO-F	TCCATGGAAACTGGTGTTGTGC	qRT-PCR
CO-R	TTGCAGGGTCAGGTTGTTGCTC	qRT-PCR
FUL-RT-F	GAGAGGGAGAAGAAAACGGGTCAG	qRT-PCR
FUL-RT-R	CTCTCCCCCAACTCTCTCCAC	qRT-PCR
SOC1F	TGAGGCATACTAAGGATCGAG	qRT-PCR
SOC1R	GCGTCTCTACTTCAGAACTTGGGC	qRT-PCR
AtTUB2-F	GAGCCTTACAACGCTACTCTGTCTGTC	qRT-PCR
AtTUB2-R	ACACCAGACATAGTAGCAGAAATCAAG	qRT-PCR

Note: F, forward primer, R, reverse primer

Table S2. Combinations of primer pair used to amplify subsections of gDNA of *TaSPL16* homoeologs in wheat.

Primer pair #	**PCR products from different subgenome (bp) ***		
	A	B	D
For multi-subgenome subsection amplification			
gF1-gR1	1194	1224	—
gF2-gR2	—	—	—
gF3-gR3	1570	1503	—
gF4-gR4	1572	—	1561
For the missing subsection in A subgenome			
gF2/gAR2	1244	—	—
For the missing subsections in B subgenome			
gF2/gBR2	—	1228	—
gBF4/gBR4	—	1561	—
For the remaining subsections in D subgenome			
gDF3/gDR3	—	—	1612
gF2/gDR2	—	—	1632
gDF1/gDR1	—	—	1456
Assembled full length sequences	5061	4824	5229

# Primer pairs for multi-subgenome subsection amplification were designed based on alignment of the *TaSPL16-cDNA1* sequence cloned with the two contigs from A genome (Genbank accession number: AOTI010433783) and D genome (Genbank accession number: AOCO010294339); Primer pairs for the missing subsection in special subgenome (A, B, or D subgenome) were designed based on alignments of the two contigs and the sequenced fragments adjacent to it.

* "—" represents no PCR product.

Table S3. Identity of nucleotide sequence or amino acid sequence among three *TaSPL16* homoeologs in wheat.

Alignment levels	Identity of three *TaSPL16* homoeologs with each other		
	TaSPL16-A/B	*TaSPL16-A/D*	*TaSPL16-B/D*
Genomic DNA sequence	96.0%	96.7%	96.4%
cDNA sequence	96.2%	98%	96.6%
Amino acid sequence	93.5%	95.4%	94.5%

Table S4. *In silico* chromosome mapping of *TaSPL16* homoeologs based on the chromosome-based draft sequence of hexaploid bread wheat genome.*

Genomic DNA (length)	Identity / cover range	Contig number.	Contig size (bp)	Chromosome bin
TaSPL16-A (5061 bp)	99.7% / 1524-4875	IWGSC_CSS_7AS_scaff_4204928	3351	7AS
TaSPL16-B (4824 bp)	97.1% / 442-4860	IWGSC_CSS_7BS_scaff_3156641	7070	7BS
TaSPL16-D (5229 bp)	99.8% / 642-5298	IWGSC_CSS_7DS_scaff_3852833	5229	7DS

* International Wheat Genome Sequencing Consortium (IWGSC). (2014). A chromosome-based draft sequence of hexaploid bread wheat (*Triticum aestivum*) genome, Science 345, 1251788. doi: 10.1126/science.1251788.

Table S5. *TaSPL16* homoeologs found in the fully annotated reference genome of wheat cultivar Chinese Spring. *

TaSPL16 homoeolog	Gene ID	Transcript ID	Chr. location
TaSPL16-7A	TraesCS7A01G260500	TraesCS7A01G260500.1	7A: 252,715,654-252,720,713
TaSPL16-7B	TraesCS7B01G158500	TraesCS7B01G158500.1	7B: 214,070,890-214,075,715
TaSPL16-7D	TraesCS7D01G261500	TraesCS7D01G261500.1	7D: 237,410,697-237,415,925

* International Wheat Genome Sequencing Consortium (IWGSC). (2018). Shifting the limits in wheat research and breeding using a fully annotated reference genome. Science 361, eaar7191. doi: 10.1126/science.aar7191.

2. Supplementary Figures

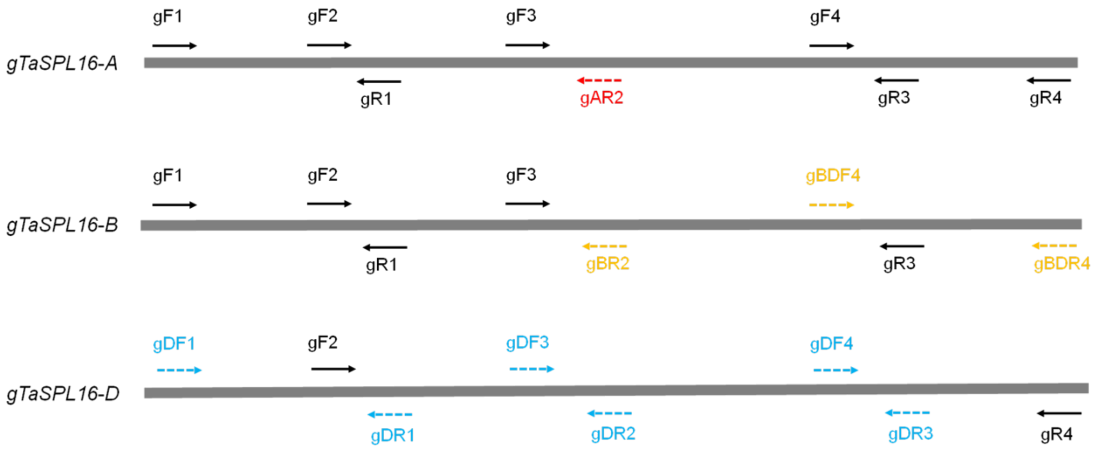

Figure S1. Schematic diagram of subsection cloning *TaSPL16* homoeologs in wheat. Gray long rough lines indicate three *TaSPL16* homoeologs (*gTaSPL16-A*, *gTaSPL16-B*, and *gTaSPL16-D*); the solid lines with arrow refer to the common primers used for subsection cloning of three *TaSPL16* homoeologs. Whilst the dotted lines with arrow indicate the specific primers only used for cloning the subsection in one or two special subgenomes, red, yellow and blue dotted lines representing specific primers for *gTaSPL16-A*, *gTaSPL16-B* and *gTaSPL16-D*, respectively.

```
  1 ctggcttgcgctgtacgtgtcctccgcgcggcagggccggctacgtgagtgcgcatcgcg
 61 gcatggactgggatctcaagatgccgcccggcgcctgggacctcaccgagctggagaacg
       M  D  W  D  L  K  M  P  P  G  A  W  D  L  T  E  L  E  N
121 acgcggccgctgcgccggcggctgcccaggcgtcggccggcggcattgctaatgcggccg
    D  A  A  A  A  P  A  A  A  Q  A  S  A  G  G  I  A  N  A  A
181 gccggccggagtgctccgtggacctgaagctcggcgggctcggcgagtgcggcgctgctc
    G  R  P  E  C  S  V  D  L  K  L  G  G  L  G  E  C  G  A  A
241 cggacagccgcggcctcggcaaagcgccggccgaggcggcgtcctcggcgtcggcgccca
    P  D  S  R  G  L  G  K  A  P  A  E  A  A  S  S  A  S  A  P
301 gcgcggcgaagcggccgcgcgcgtcgtcgggagggggaggttggagcggcgcggggcagc
    S  A  A  K  R  P  R  A  S  S  G  G  G  G  W  S  G  A  G  Q
361 agcagtgtccgtcgtgcgcggtggacgggtgtagggcggacctgagcaagtgccgcgact
    Q  Q  C  P  S  C  A  V  D  G  C  R  A  D  L  S  K  C  R  D
421 accatcgccggcacaaggtgtgcgaggcgcactccaagacccccgtcgtcaccgtcgccg
    Y  H  R  R  H  K  V  C  E  A  H  S  K  T  P  V  V  T  V  A
481 gccgcgagatgcgcttctgccaacagtgcagcaggtttcacctgcttacggagtttgatg
    G  R  E  M  R  F  C  Q  Q  C  S  R  F  H  L  L  T  E  F  D
541 aggccaaacgcagctgtagaaagcgtcttgatgggcacaaccgtcgccgcaggaaaccgc
    E  A  K  R  S  C  R  K  R  L  D  G  H  N  R  R  R  R  K  P
601 agccagatgtgatgaattctgcaagttttatgacaagtcaacaaggaacaaggttttcat
```

```
     Q  P  D  V  M  N  S  A  S  F  M  T  S  Q  Q  G  T  R  F  S
 661 catttccaactccaagaccggagcaaaactggccagggatcattaaaactgaggagaacc
     S  F  P  T  P  R  P  E  Q  N  W  P  G  I  I  K  T  E  E  N
 721 cctattacgcacatcaactccctctaggcatcagcaacaggcagcattttggtggctctg
     P  Y  Y  A  H  Q  L  P  L  G  I  S  N  R  Q  H  F  G  G  S
 781 cgtcgacttacgccaaagaaggacggcgatttcctttcctgcaggaaggcgaaataaact
     A  S  T  Y  A  K  E  G  R  R  F  P  F  L  Q  E  G  E  I  N
 841 ttgccactggtgtggcacttgagccttcagtgtgccaaccgctcctcaagacggtagctc
     F  A  T  G  V  A  L  E  P  S  V  C  Q  P  L  L  K  T  V  A
 901 ctcccgagagcagcagcagcagcagcaagatgttctctgatgggctgactccagtgctgg
     P  P  E  S  S  S  S  S  S  K  M  F  S  D  G  L  T  P  V  L
 961 actcagactgtgctctctctcttctgtcagctccggcaaactcctctggtatcgatgttg
     D  S  D  C  A  L  S  L  L  S  A  P  A  N  S  S  G  I  D  V
1021 gcccgatggtccaacagactgaacacatccccattgcccagcctctgttctccaacctgc
     G  P  M  V  Q  Q  T  E  H  I  P  I  A  Q  P  L  F  S  N  L
1081 aattcagcagctcgtcctggttctcgcgcacccaggcttccaccggtaccgtctcagcga
     Q  F  S  S  S  S  W  F  S  R  T  Q  A  S  T  G  T  V  S  A
1141 ctggattttcctgccctgtgggggaaaatgagcaactgaacaatgtgctaagctcggaca
     T  G  F  S  C  P  V  G  E  N  E  Q  L  N  N  V  L  S  S  D
1201 gcaatgacttgaactacaatgggatatttcatgtcggcggtgaaggctcgtcggacggcg
     S  N  D  L  N  Y  N  G  I  F  H  V  G  G  E  G  S  S  D  G
1261 ccccgccatctctgcccttcccgtggcagtaggtttcag
     A  P  P  S  L  P  F  P  W  Q  *
```

Figure S2. Nucleoid acid and deduced amino acid sequences of *TaSPL16-cDNA1*. The start codon ATG and stop codon TAG are indicated in red. The miR156 binding site that consists of 21 nucleotides in the cDNA sequence is indicated in pink. The underlined sections indicate conserved SBP domain, in which the two zinc binding sites (Cys-Cys-Cys-His and Cys-Cys-His-Cys) are marked in yellow and blue, respectively, and the NLS in green.

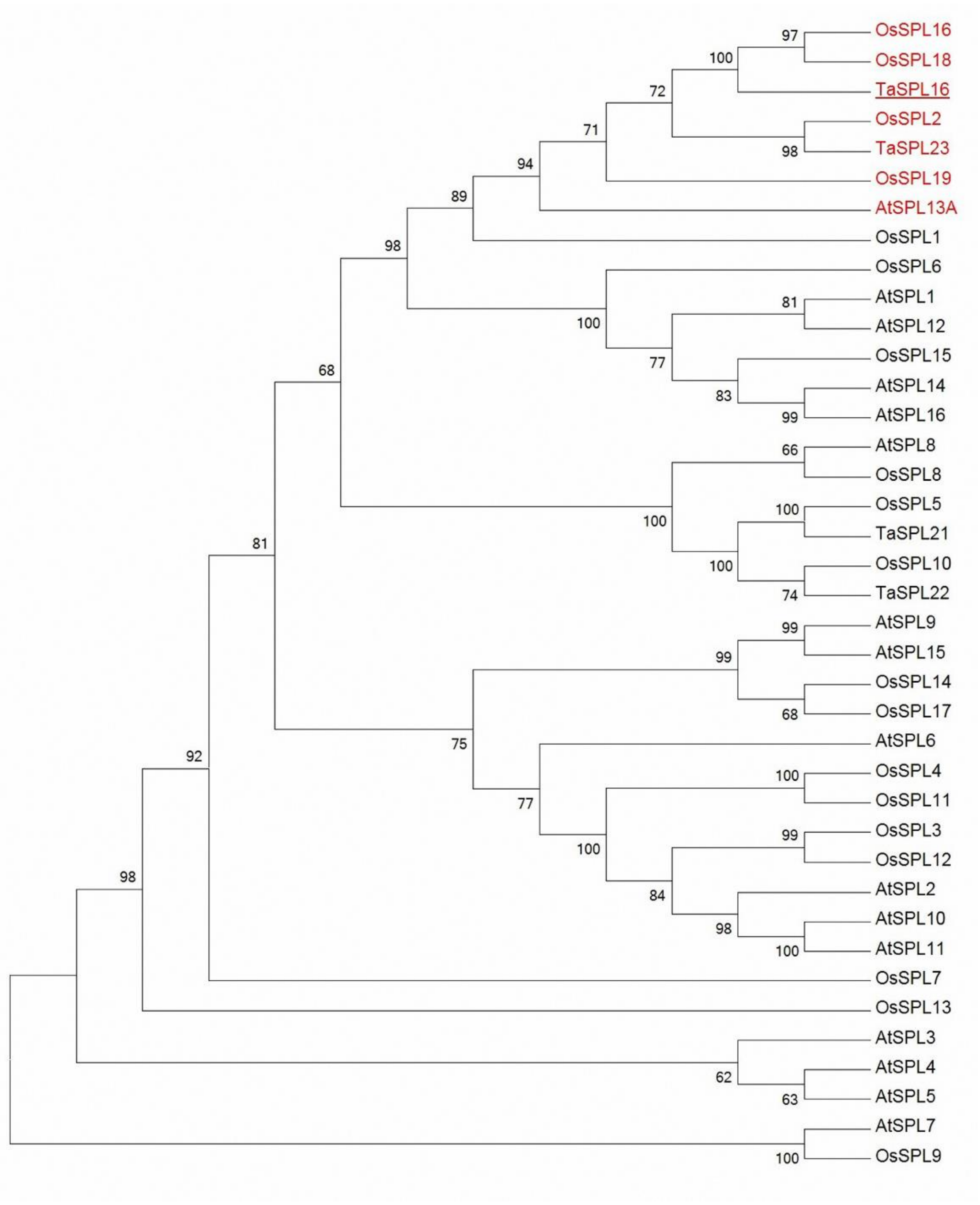

Figure S3. Genetic relationship of *TaSPL16* with 16 *SPL*s in *Arabidopsis* and 19 *SPLs* in rice. To further understand the genetic relationship of TaSPL16 and TaSPL23 with OsSPL16 in rice or AtSPL13A in *Arabidopsis*, amino acid sequences of SBP domain from these proteins were download and aligned by ClustalW, and MEGA5.05 was used to developed a neighbor-joining tree with 1,000 bootstrap replicates. TaSPL16 and its homologous in the same group were remarked in red. The bootstrap values in trees are shown next to the branches associated taxa clustered together.

```
gTaSPL16-A   CTGGCTTGCGCTGTACGTGTCCTCCGCGCGCCAGGGCCGGCTACGTGAGCGCGCATCGCG   60
gTaSPL16-B   CTGGCTTGCGCTGTACGTGTCCTCCGCGCGGCAGGGCCGGCTACGTGAGTGCGCATCGCG   60
gTaSPL16-D   CTGGCTTGCGCTGTACGTGTCCTCCGCGCGCCAGGGCCGGCTACGTGAGTGCGCACCGCG   60
```

```
gTaSPL16-A    GCATGGACTGGGATCTCAAGATGCCGCCCGGCGCATGGGACCTCGCCGAGCTGGAGGGCG    120
gTaSPL16-B    GCATGGACTGGGATCTCAAGATGCCGCCCGGCGCCTGGGACCTCACCGAGCTGGAGAACG    120
gTaSPL16-D    GCATGGACTGGGATCTCAAGATGCCGCCCGGCGCATGGGACCTCGCCGAGCTGGAGAGCG    120
                ******************************** ********* ***********  **

gTaSPL16-A    ACGCG---------CCGGCGGCGGGCGGGCAGGCGTCGGCCGGTGACATTGCTAATGCGG    171
gTaSPL16-B    ACGCGGCCGCTGCGCCGGCGGCTGCC---CAGGCGTCGGCCGGCGGCATTGCTAATGCGG    177
gTaSPL16-D    ACGCGGCCGCCGCGCCGGCGGCGGGCGGGCAGGCGTCGGCCGGTGGCATTGCTAATGCGG    180
              *****         ******** * *   ************** *.**************

gTaSPL16-A    CCGGCCGGCAGGAGTGCTCCGTGGACCTGAAGCTCGGCGGGCTCGGCGAGTGCGGCGCTG    231
gTaSPL16-B    CCGGCCGGCCGGAGTGCTCCGTGGACCTGAAGCTCGGCGGGCTCGGCGAGTGCGGCGCTG    237
gTaSPL16-D    CCGGCCGGCAGGAGTGCTCCGTGGACCTGAAGCTCGGCGGGCTCGGCGAGTGCGGCGCTG    240
              *********.**************************************************

gTaSPL16-A    CTCCGGACAGCCGCGGCCTCGGCAAGGCGCCGGCCGAGGCGCCGTCCTCG----------    281
gTaSPL16-B    CTCCGGACAGCCGCGGCCTCGGCAAAGCGCCGGCCGAGGCGGCGTTCTCGGCGTCG----    293
gTaSPL16-D    CTCCGGGCAGCCGCGGTCTCGGCAAGGCGCCGGCCGAGGCGGCGTCCTCGGCGTCGGCGT    300
              ******.********* ********.*************** *** ****

gTaSPL16-A    --GCGCCCAGCGCGGCGAAGCGGCCGCGCGCGTCGTCGGGAGGGGGAGGCGGGAGCGGGA    339
gTaSPL16-B    --GCGCCCAGCGCGGCGAAGCGGCCGCGCGCGTCGTCGGGAGGGGGAGGTTG------GA    345
gTaSPL16-D    CCGCGCCCAGCGCGGCGAAGCGGCCGCGCGCGTCGTCGGGAGGGGGAGGAGGGAGCGGGA    360
                ***********************************************  *      **

gTaSPL16-A    GCGGCGCGGGGCAGCAGCAGTGCCCGTCGTGCGCGGTGGACGGGTGCAGGGCGGACCTGA    399
gTaSPL16-B    GCGGCGCGGGGCAGCAGCAGTGTCCGTCGTGCGCGGTGGACGGGTGTAGGGCGGACCTGA    405
gTaSPL16-D    GCGGCGCGGGGCAGCAGCAGTGCCCGTCGTGTGCGGTGGACGGGTGCAAGGCGGACCTGA    420
              ********************** ******** ************** *.***********

gTaSPL16-A    GCAAGTGCCGCGACTACCATCGCCGGCACAAGGTGTGCGAGGCGCACTCCAAGACCCCCG    459
gTaSPL16-B    GCAAGTGCCGCGACTACCATCGCCGGCACAAGGTGTGCGAGGCGCACTCCAAGACCCCCG    465
gTaSPL16-D    GCAGGTGCCGCGACTACCATCGCCGGCACAAGGTGTGCGAGGCGCACTCCAAGACCCCCG    480
              ***.********************************************************

gTaSPL16-A    TCGTCACCGTCGCCGGCCGCGAGATGCGCTTCTGCCAACAGTGCAGCAGGTAATCATTTC    519
gTaSPL16-B    TCGTCACCGTCGCCGGCCGCGAGATGCGCTTCTGCCAACAGTGCAGCAGGTAATTATTTC    525
gTaSPL16-D    TCGTCACCGTCGCCGGCCGCGAGATGCGATTCTGCCAACAGTGCAGCAGGTAATTATTTC    540
              ********

gTaSPL16-A    ACCCGCCAATTCCAACCAAGTACGCTACTTGAATCAAATCGTAGCTCTTCTTTCGTTTTT    579
gTaSPL16-B    ACCCGCCAATTCCAACCAAGTACGCTACTTAAATCAAATCGTAGCTCTTCTTTTTCTTTT    585
gTaSPL16-D    ACCCGCCAATTCCAACCAAGTGCGCTACTTGAATCAAATCGCAGCTCTTCTTTTTTTTTG    600

gTaSPL16-A    TGCATCATCATTCTCTTCGCTC--------CCTTCATCGTCCATCCATGAGTCGATCAAT    631
gTaSPL16-B    TTGCGTATCATTCTCTTCGCTCATCCATT-CCTTCATCGTCCATCCAT----CAATCAAT    640
gTaSPL16-D    AATC--ATCATTCTCTTCGCTCATCCGTTGCCTCCATCGTGCATCCATGAGTCGGTCAAT    658

gTaSPL16-A    GATCAATTCACCACCGTGACATTAT---TAGTTTGTTTTT----------------CCTT    672
gTaSPL16-B    GATCAATACACTACCGTCACATTATTACTAGTTTGTTTTTTCCTCTCATTTTTTTTTTCT    700
gTaSPL16-D    GATCAATTCACCACCGTGACATTAT---TAGTTTGTTTTTTTTCCT----TCTTTTCCCT    711

gTaSPL16-A    TTTTTTGAGCACAATTCGGCCTGTCTCGTATCGAGGATGCACTTCTTTACCTTTCCCGGC    732
gTaSPL16-B    TTGTTTGAGCACAATTCGGCCTGTCTCGTATCGAGGATGCACTTCTTTACCTTTCCCGGC    760
```

gTaSPL16-D	TTGTTTGAGCACAACTCGGCCTGTCTCGTATCGAGGATGCACTTCTTTACCTTTCCCGGC	771
gTaSPL16-A	ACCATGCCTGGCCTCTCATTTGCTCCTGTCACCATTTATCATGGCAGCTTGTGTTACCTG	792
gTaSPL16-B	ATCATGCCTGGCCTCTCATTTGCTCCTGTCACCCTTTATCATGGCAGCTTGTGTTACCTG	820
gTaSPL16-D	ATCATGCCTGGCCTCTCATTTGCTCCTGTCACCCTTTATCATGGCAGCTTGTGTTACCTG	831
gTaSPL16-A	AATTATTGCGACGCTTTTTCTTTCGTTTTTTTCTTTCCTACTACCGCTTTTACGCTGCTC	852
gTaSPL16-B	AATTATTGCAACGCTTTTTCTTTCCG-TTTTTTTTTCCTACTACCGCTTTTACGCAGCTC	879
gTaSPL16-D	AATTATTGCAACGCTTTTTCTTTCGG-TTTTTTT---CTACTACCGCTTTTACGCTGCTC	887
gTaSPL16-A	AGAGCTTGTTGGACTGCACCAATTTGCTCCTCCATCATCAATTCCTATCAGCACAGCTTT	912
gTaSPL16-B	AGAGCTTGTTGGACTGCACCAATTTGCTCCTCCAT---CAATTCCTATCAGCACAGCTTT	936
gTaSPL16-D	AGAGCTTGTTGGACTGCACCAATTTGCTCCTCCATCATCAATTCCTATCAGCACAGCTTT	947
gTaSPL16-A	TGCACTGCTCTGAATCGCCCCACCACCTTTTCTGAATCCTGGCCCCATTCATGAGAATTC	972
gTaSPL16-B	TGCACTGCTCTGAATCGCCTCACCACCTTTTCTGAATCCTGGCCCCATTCATGAGAATTC	996
gTaSPL16-D	TGCACTGCTCTGAATCGCCCCACCACCTTTTCTGAACCCTGGCCCCATTCATGAGAATTC	1007
gTaSPL16-A	AGCACTGCACAAAAGGCTACATTTGCTCTCCATCTGCCAAAGATCAAGATCACATC----	1028
gTaSPL16-B	AGCACTGCACAAAAAGCTACATTTGCTCTCCATCTGCCAAAGATCAAGATCACATCCACA	1056
gTaSPL16-D	AGCACTGCACAAAAAGCTACATTTGCTCTCCATCTGCCAAAGATCAAGATCACATCCACA	1067
gTaSPL16-A	--GGCACCCAATGATCATCAATCGATCAGCCTACTGAATCTGTCCGATTTGCATTTGACG	1086
gTaSPL16-B	CAGGCACCCAATGATCATCAATCGATCAGCCTACTGAATCTGTTTGATTTGCAGTTGACG	1116
gTaSPL16-D	CAGGCACCCAATGATCATCAATCGATCAGCCTACTGAACCTGTCCGATTTGCATTTGACG	1127
gTaSPL16-A	TGTTAAATCTGTTCCGTGTTAATTGGAGTTGTTTTTCTCGGTGCGCGACACTGTTCTGAT	1146
gTaSPL16-B	AGTTAAATCTGTTCCGTGTTAATCGGAGTTGTTTTTCTCGGTGCGCG--ACTGTTCTGAT	1174
gTaSPL16-D	TGTTAAATCTGTTCCGTGTTAATTGGAGTTGTTTTTCTCGGTGCGCGACACTGTTCTGAT	1187
gTaSPL16-A	ATCGAGTTCAGCAACGTGCCGCCTCGTGATCGATGATAACCCGGACGGCCGGACGGACGC	1206
gTaSPL16-B	ATCGATTTCAGCAACGTGCCGCCTCGTGATCGATGATAACCCGGACGGACAGACGGACGC	1234
gTaSPL16-D	ATCGATTTCAGCAACGTGCCGCCTCGTGATCGATGATAACCCGGACG--------GACGC	1239
gTaSPL16-A	CTGATATGATTTGCCCGCGCCTCCTTTTTCATGTGCAAAATAGGTGGATCCATGTGCATT	1266
gTaSPL16-B	CTGATATGATTTGCCCGCGCCTCCTTTT---------------------CCATGTGCATT	1273
gTaSPL16-D	CTGATATGATTTGCCCGCGCCTCCTTTTTCATGTGCAAAATAGGTGGATCCATGTGCATT	1299
gTaSPL16-A	CTTGTGCTTT-------------CCTGGCCGCGACAGCATGTTTTGTGTGGGTCTGGGAC	1313
gTaSPL16-B	GTTCTGCTTTTTCCTGTGGATTTCCTGGCCGCGACAGCATGTTTTGTGTGGGTCTGGGAC	1333
gTaSPL16-D	GTTGTGCTTTTTCCTGTGGATTTCCAAGACGCGACAGCATGTTTTGTGTGGGTCTGGGAC	1359
gTaSPL16-A	TGTCAATTACTTAAACATGGCAGGTATATCCGGCCTGCTCCATTCATCACGCGGCGGCAA	1373
gTaSPL16-B	TGTCAATTACTTAAGCATGGCAGGTATATCCGGCCTGCTTCATTCATCACGCG---GCAA	1390
gTaSPL16-D	TGTCAATTACTTAAGCATGGCAGGTATATCCGGCCTGCTTCACTCATCACACC---GCAA	1416
gTaSPL16-A	GAACTGGCCTACCACATCCGAGCCCTCTGTGTCAATGGAAAAGGCCATGCTAACTCCCAA	1433
gTaSPL16-B	GAACTGGCCTACCACATCCGAGCCCTCTGTTTCAAAGGAAAGGGCGATGCTAACTCCCAA	1450
gTaSPL16-D	GAACTGGCCTACCACATCCGAGCCCTCTGTTTCGAAGGAAAAGGCGATGCTAACTCCCAA	1476
gTaSPL16-A	ACCTAGAAAGTTTATAGTACTACTACACAAGTGTTTACTCTGCAAAATGCAGTGTGGACT	1493
gTaSPL16-B	ACCTAGAAAGTTTATAGTACCACTACACAAGTGCTTACTCTGCAAAATGCAGTGTGGACT	1510
gTaSPL16-D	ACCTAGAAAGTTTATAGTACCACTACACAAGTGCTTACCCTGCAAAATGCAGTGTGGACT	1536
gTaSPL16-A	T-GTAGACTGTGCGTGCAGTGTGGATGACTCGAGCCATTTCTGTACGATATAGGAGGAG-	1551

```
gTaSPL16-B   TTGTGGACTGTGG----AGTGTGGATGACTCGAGCCATTTCTGTACGATATAGGAGGAGG   1566
gTaSPL16-D   T-GTGGACTGTGG----AGTGTGGATGACTCGAGCCATTTCTGTACGATACAGGAGGAGG   1591

gTaSPL16-A   ------------------------------TATTTTCTAAGGCTGGCCAGGCTTGCAACC   1581
gTaSPL16-B   ACCATGCGTGCATGTTCACAGATACATGTATATTTTCTAAGGCTGGCCAGGCTTGCAACC   1626
gTaSPL16-D   GCCATGCGTGCATGTTCACTGATACATGTATATTTTCCAATGCTGGCCAGGCTTGCAATC   1651

gTaSPL16-A   AGCCGAGGGCCTGAAAGTTGTTCATGTTTTAGTTTCATTAAAAGGTTAATGGTAGTGTCA   1641
gTaSPL16-B   GGCCGAGGGCCTGAAAGTTGTTCATGTTTTAGTTTCAT----------------------   1664
gTaSPL16-D   AGCCGAGGGCCTGAAAGTTGTTCATGTTTTAGTTTCATTAAAAGGTTAATGGTAGTGCCA   1711

gTaSPL16-A   ACTTCCGCAAAAATATAAATGGTAGTGCCAACTGAAAATGATG-----------------   1684
gTaSPL16-B   ------------------------------------------------------------
gTaSPL16-D   ACTTTCGCAAAAATACAAATGGTAGTGCCAACTGAAAATGATGTATCTTAAATTATTTTT   1771

gTaSPL16-A   ------------------------TATCCTAAATATTAAAAGGCGACACCAACCCATATAG   1721
gTaSPL16-B   ----------------------------TAAATATTAAAAGGCGACACCAACCCATATAG   1696
gTaSPL16-D   ATTTTTATTTTTGCGGAAAGATGTATCTTAAATATTAAAAGGCGACACCAACCCATATAG   1831

gTaSPL16-A   CCTGATCTAGCGATTCGGTTGACGAGCATGAGAAAAATGAGATCTCTAGGCTCTTGCATC   1781
gTaSPL16-B   CGTGATCTAGTGATTCGGTTGATGAGCATGAGAAAAATGAGATCTCTAGGCTCTTTCATC   1756
gTaSPL16-D   CGTGATCTAGTGATTCGGTTGATGAGCATGAGAAAATTGAGATCTCTAGGCTCTTGCATC   1891

gTaSPL16-A   CTGATCAGTTTGGACTTTGGACTTCATGGGAGCCCTTATCGCAAGTTCTTTT--------   1833
gTaSPL16-B   CTGATCAGTTTGGACTTTGGACTTCATGGGAGCCCTTATCACAAGTTCTTTT--------   1808
gTaSPL16-D   CCGATCAGTTTGGACTTTGGACTTCGTGGGAGCCCTTATCGCAAGTTCTTTTTGTTGAAA   1951

gTaSPL16-A   ------------------------------------------------------------
gTaSPL16-B   ------------------------------------------------------------
gTaSPL16-D   TATATTGCCCGCCTTCCTCCATCAGTTCGGACTTTTGGATGAGTTGGCTGGAGCATGAAT   2011

gTaSPL16-A   ------------------------------------------------------------
gTaSPL16-B   ------------------------------------------------------------
gTaSPL16-D   TCAATATGGTATCAGAGCCAAGAGGTCTTGAGTTCAAGACCCTGCCAACGCAGTATTATA   2071


gTaSPL16-A   ------------------------------------------------------------
gTaSPL16-B   ------------------------------------------------------------
gTaSPL16-D   AAAAAAACGATTCTGTGGCCTACATCAAACCCACGTCTAAGGACTAAAATAGCCTAGACG   2131

gTaSPL16-A   ------------------------------------------------------------
gTaSPL16-B   ------------------------------------------------------------
gTaSPL16-D   TGAGGGAGAGTGTTGAAATATATTGCCCGCCTCCCTCCATCAGTTCGGACTTTTGGATGA   2191

gTaSPL16-A   -----------------------------AATTAAGATAAACAATTTCTTGAATGTGCAG   1864
gTaSPL16-B   -----------------------------AATTAAGATAAACAATTTCTTGAATGTGAAG   1839
gTaSPL16-D   GTTGGCTGGAGCATGAATTCAATACTTTTAATTAAGATAAACAATTTCTTGAATGTGCAG   2251

gTaSPL16-A   GGGTCAAATCCTACTTATCATGTTAATGTTTCATGGTCTATGTTGTGGGGATAGCATTTC   1924
gTaSPL16-B   GCGTCAAATCCTACTTATCATGTTAATGTTTCATGGTCTATGTTGTGGGGATAGCATTTC   1899
gTaSPL16-D   GGGTCAAATCCTACTTATCATGTTAATGTTTCATGGTCTATGTTGTGGGGATAGCATTTC   2311

gTaSPL16-A   TAGGTCATT-AAGCATCGTGCGGTCGTCATCAGAAAAGAAAAAGAAGGGTATTATATCGT   1983
gTaSPL16-B   TAGGTCATTTAAGTATCGTGCGGTCGTCATCAGAAAAGAAAAAGAAGGGTATTATATCGT   1959
```

gTaSPL16-D TAGGCCATTTAAGTATCGTGCGGTCGTCATCAGAAAAGAAAAAGAAGGATATTATATCGT 2371

gTaSPL16-A GATAATATCTCAAACGTTAGTGTCAGCGAAATAACATATGATAATTTGCTTGTTTCAGGT 2043
gTaSPL16-B GATAATATCTCAAACGTTAGTGTCAGAGAAATAACATATGATAATTTGCTTGTTTCAGGT 2019
gTaSPL16-D GATAATATCTCAAACGTTAGTGTCAGCGAAATAACATATGATAATTTGCTTGTTTCAGGT 2431

gTaSPL16-A GCTTTGGGACCACTTGACTCTGTTTTTGTCTGAGTTGCACTTCTGCAA--ATCCTTCTAG 2101
gTaSPL16-B GCTTTGGGACCACTTGACTCTGTTTTTGTCTGAGTTGCACTTCTGCAATTATCCTTCTAG 2079
gTaSPL16-D GCTTTGGGACCACTTGACTCTGTTTTTGTCTGAGTTGCACTTCTGCAATTATCCTTCTAG 2491

gTaSPL16-A GATAGCTTCGCTGTATTTGTTTTCATGCATTAGCCAGAAGACTGAGATAATCACATTCTA 2161
gTaSPL16-B GATAGCTTCCCTGTATTTGCTTTCATGCATTAGCCAGAAGACTGAGATAATCACATTCTA 2139
gTaSPL16-D GATAGCTTCGCTGTATTTGTTTTCATGCATTAGCCAGACGACTGAGATAATCACATTCTA 2551

gTaSPL16-A TGAGCAGAACTAATTGGATCAAATTTGTCACATTTTTTATGTGAGGGAAAATAATAGAGA 2221
gTaSPL16-B TGAGCAGAACTAATTGGATCAAATTTGTCACATTTTTTGTGTAAGGAAAAATAACAGAG- 2198
gTaSPL16-D TGACCAGAACTAATTGGATCAAATTTGTCACATTTTTTATGTAGG-AAAAATAACAGAGA 2610

gTaSPL16-A GATTCAGTTCTCCATTTTTCAAATGTAGAATCGATAGTCTTACTGTATATCCTCAACTCG 2281
gTaSPL16-B -ATTCAGTTCTCCATTTTTCAAATGTAGAATCGATAGTCTTACTGTTTATCCTCTACTCG 2257
gTaSPL16-D GATTCAGTTCTCCATTTTTCAAATGTAGAATCGATAGTCCTACTGTATATCTTCTACTCG 2670

gTaSPL16-A AAAACCTGAACCATCCATAGTATTGTTATTTCGGGTTGTAGTCTCTTGCATTATTTCTTA 2341
gTaSPL16-B G-AACCTGAACCATCTATAGTATTGTTATTTCGGGTTGTAGTCTCTTGCATTATTTCTCA 2316
gTaSPL16-D G-AACCTGAACCATCCATAGTATTGTTATTTCGGGTTGTAGTCTCTTGCATTATTTCTTA 2729

gTaSPL16-A CATTTGCAATATTTTCGTATCTTGGTCCACTTTAAAGCACAGTTGTGTTTATTCTCATTG 2401
gTaSPL16-B CATTTGCAATATTTTTGTACCTTGGTCCACTTTAAAGTACAGTTGTGTTTATTCTCATTT 2376
gTaSPL16-D CGTTTGCAATATTTTTGTATCTTGGTCCACCTTAAAGTATAGTTGTGTTTATTCTCATTT 2789

gTaSPL16-A TAATCTTGCTGAGCATAGCTGGTGATTCTATGGACCTCTACCTTTATACCTTTATAACTT 2461
gTaSPL16-B TAATCTTGCTGAGCATATCAGGTGATTCTATGGACTTCTACCTTTATACCTTTATAACTT 2436
gTaSPL16-D TAATCTTGCTGAGCATAGCAGGTGATTCTATGGACCTCTACCTTTATACCTTTATAACTT 2849

gTaSPL16-A GATAGGCTGCTAATGTGTTCCTCATCATGTTATTGCCTTGATACATGAATATCTTACCCA 2521
gTaSPL16-B CGTAAGCTGCTAATGTGTTCCTCGTCATGTTATTGCCTTGATACATGAATATCTTACCCA 2496
gTaSPL16-D GATAGGCTGCTAATGTGTTCCTCATCATGTTATTGCCTTGATACATGAATATCTTACCCA 2909

gTaSPL16-A GTGGGGACAGTTTCCAATCATTGATTGATCCGGCAGTTAGTGCAGACTTTATAAATTAAG 2581
gTaSPL16-B GTGGGGACAGTTTCCAATCATTGACTGATCCGGCAGTTAGTGCAGACTTTATAAATTAAG 2556
gTaSPL16-D GTGGGGATAGTTTCCAATCATTGATTGATCCGGCAGTTAGTGCAGACTTTATAAATTAAG 2969

gTaSPL16-A TGCGCGCATTGCCCAGCAGACTTGCATCCTTCTAGACATATGG-TTACAGACACACTATA 2640
gTaSPL16-B TGCGCGCATTGCCCAGCAGACTTGCGTCCTTCTAGACATACGGTTAACAGACACACTATA 2616
gTaSPL16-D TGCGCGCATTGCCCAGCAGACTTGCATCCTTCTAGACATATGG-TTACAGACACACTATA 3028

gTaSPL16-A CTGTGGTCCATGTTCAACAGGCATAGCAATATTTTTGTCTGCTGTTGACAAATCACAGGT 2700
gTaSPL16-B CTGTGGTCCATGTTCAACTGGCATCGCAATATTTTTGTCTGCTGTTGACAAATCTCAGGT 2676
gTaSPL16-D CTGTGGTCCATGCTCAACAGGCATCGCAATATTTTTGTCTGCTGTTGACAAATCTCAGGT 3088

gTaSPL16-A GCAATATTTTTATCAAAGATGAAGGCTCCCAAGCACTGAAAGGAGTGATTGACAGATGAA 2760
gTaSPL16-B ACAATATTTTTATCTAAGATGAAGGCTCCCAAGCACTGAAAGGAGTGATTGACAGATGAA 2736
gTaSPL16-D ACAATATTTTTATCTAAGATGAAGGCTCCCAAGCACTGAAAGGAGTGATTGACAGATGAA 3148

```
gTaSPL16-A   TTAAGTGGTCCCTTTGTGTACTGTACATTTTATATTCCAACAGTATGTGTGAAGAATGTT   2820
gTaSPL16-B   TTAAGTGGTCCCTTTGTGTACTGTATATTTTATATTCCAACAGTATGTGTGAAGAATATT   2796
gTaSPL16-D   TTAAGTGGTCCCTTTGTGTACTGTACATTTTATATTCCAACAGTATGTGTGAAGAATATT   3208

gTaSPL16-A   AAGTATTCTGACAATATGTAGTTTGGTGATTTAGCGAGAGCATATTTTT-ATACTTCGAA   2879
gTaSPL16-B   AAGTATTCTGACATTATGTAGTTTGGTGATTTAGCGAGAGCATATTTTT-ATACTTCGAA   2855
gTaSPL16-D   AAGTATTCTGACAATATGTAGTCTGGTGATTTAGCGAGAGCATATTTTTAATACTTCGAA   3268

gTaSPL16-A   ACATGTGTCTAACTACCTAGGAGCATCTTATCTTGTTATAAAGTCATCCTAGGACAAGGA   2939
gTaSPL16-B   ACACGTGTCTAACTACCTAGGAGCATCTTATCTTGTTATAAAATCATCCTAGGACAAGGA   2915
gTaSPL16-D   ACACGTGTCTAACTACCTAGGAGCATCTTATCTTGTTATAAAATCATCCTAGGACAAGGA   3328

gTaSPL16-A   ATACCTTATTTAGTTTTCAACAAAGACTAGCAATATTGTTTCTTAAGACTTAAGAGCTAA   2999
gTaSPL16-B   ATATCTTATTTAGTTTTCAACAAAGACTAGAAATATTGTCTCTTAAGACTTAAGAGCTAA   2975
gTaSPL16-D   ATATCTTATTTAGTTTTCAACAAAGACTAGCAATATTGTCTCTTAAG-------AGCTAA   3381

gTaSPL16-A   GGAAAAAAGCTACATCCTATTTAAATCTTAAAAGTGGTCGGACCCTTCCCTGGACCCTGC   3059
gTaSPL16-B   GGAAATAACCTACATCCTATT---------------------------------------   2996
gTaSPL16-D   GGAAAAAAGCTACATCCTATT---------------------------------------   3402

gTaSPL16-A   GCAAGCGGGAGCTACATGCACCGGGCTGCCTATTTAAAATCTTAGTTGCTGTGTTCTTTA   3119
gTaSPL16-B   ----------------------------------TAAAATCTTAGTTGCTGCGTTCTTTA   3022
gTaSPL16-D   ----------------------------------TAAAAGCTTAGTTGCTGTGTTCTTTA   3428

gTaSPL16-A   ATAAAAAAAGGCGCATGACACCCCAGCATTACAGAAAGCGTCAAGGATAACAGTGTGTTG   3179
gTaSPL16-B   ATAAAAAAGGGTGCATGACACCCCAGCATTACAGAAAGCGTCAAGAATAACAGTGTGTTG   3082
gTaSPL16-D   ATAAAAAAGGGCGCATGACACCCCAGCATTACAGAAAGCGTCAAGGATAACAGTGTGTTG   3488

gTaSPL16-A   TTATGTTTCAGATAAATGTTTTAAGTTATGGAACACTTTG-----TCGTTTTATTAAATC   3234
gTaSPL16-B   TTAGGTTTCGGATAAATGTTTTAAGTTATGGAACATTTGCCATCCTCGTTTTCTTAAATC   3142
gTaSPL16-D   TTATGTTTCGGATAAATGTTTAAAGTTATGGAACATTTTCCATCCTCGTTTTCTTAAATC   3548

gTaSPL16-A   CCTTTCAGATACTTGGGAGTTGATATTAGTATCTCTTTATTTTT-ACAGGAAAACTCTTG   3293
gTaSPL16-B   CCCTTCAGATACTTGGGAGTTGATATGAGTATCTCTTTATTTTTTACAGGAAAATTCTTG   3202
gTaSPL16-D   CCCTTCAGATACTTGGGAGTTGATATTAGTATCTCTTTATTTTTTACAGGAAAACTCTTG   3608

gTaSPL16-A   AGGTATTGCTTATTCAGTATTTGTAACGGTGGCTATGCTTTTGAAAGTAGCTGGAAACAC   3353
gTaSPL16-B   AGGTGTTGCTTAATAAGTATATGTAGCGGTAGCTATGTTTTTGAAAGTAGCTGGAAACAC   3262
gTaSPL16-D   AGGTATTGCTTATTCAGTATTTGTAGCGGTAGCTATGCTTTTGAAAGTAGCTGGAAACAC   3668

gTaSPL16-A   ACAAAAAAATGAGATGTTTGTAGAGCAAATTTCAGCATGTTATTACAGTTTACTATTCAGC   3413
gTaSPL16-B   ACAAAAATTGAGATGTTTGTAGACCAAATTTCAGCATGTTATTACAGTTTACTATTCAGC   3322
gTaSPL16-D   ACAAAAATTGAGATGTTTGTAGACCAAATTTCAGCATGTTATTACAGTTTACTATTCAGC   3728

gTaSPL16-A   ATTGCATTAGCTTAATTTCCTCGTGTTGTGACCTGATATTAATCCTTCATGCATCAGCAA   3473
gTaSPL16-B   ATTGCATTAGCTTAATTTCCTCATGTTGTGACCTGATATTAATCCTTCATGCATCAGCAA   3382
gTaSPL16-D   ATTGCATTAGCTTAATTTCCTCTTGTTGTGACCTGATATTAATCCTTCATGCATCAGCAA   3788

gTaSPL16-A   TAATTTGTGTCTTGATCATGAACTTTATTTCCTTAGGTTTCACCTGCTTACGGAGTTTGA   3533
gTaSPL16-B   TAATTTGTGTCTTG-TCATGAACTTTATTACCTTAGGTTTCACCTGCTTACGGAGTTTGA   3441
gTaSPL16-D   TAATTTGTGTCTTGATCATGAGCTTTATTTCCTTAGGTTTCACCTGCTTACGGAGTTTGA   3848
                                                 ************************

gTaSPL16-A   TGAGACCAAACGCAGCTGTAGAAAGCGTCTTGATGGGCACAACCGTCGCCGCAGGAAACC   3593
```

```
gTaSPL16-B   TGAGGCCAAACGCAGCTGTAGAAAGCGTCTTGATGGGCACAACCGTCGCCGCAGGAAACC   3501
gTaSPL16-D   TGAGGCCAAACGCAGCTGTAGAAAGCGTCTTGATGGGCACAACCGTCGCCGCAGGAAACC   3908
             ****.*******************************************************

gTaSPL16-A   GCAGCCAGATGTGATGAATTCTGCAAGTTTTATGACGAGCCAACAAGGTTACATTTTCAC   3653
gTaSPL16-B   GCAGCCAGATGTGATGAATTCTGCAAGTTTTATGACAAGTCAACAAGGTTACATTTTCAC   3561
gTaSPL16-D   GCAGCCAGATGTGATGAATTCTGCAAGTTTTATGACGAGTCAACAAGGTTACGTTTCCAC   3968
             ************************************.** *******

gTaSPL16-A   TACTGCTCTATATTTTAACAAGTTTTATGCCAGCCGGTTGTGGCTTTTCTTTTGGCTAGT   3713
gTaSPL16-B   TACTGCTCTATATTTTAACAG---------------------------------------   3582
gTaSPL16-D   TACTGCTCTATATTTTAATAG---------------------------------------   3989

gTaSPL16-A   TTTGTTTTTGGCCTTTTGGCTCTTTGTGAGCTTTCCTTTACCTTCGTTCGAGACTTTAAG   3773
gTaSPL16-B   ------------------------------------------------------------
gTaSPL16-D   ------------------------------------------------------------

gTaSPL16-A   ACTTTGTTGAACCTTTTTGCTTATCTATAAAGTTGGCCGTATGCATCGATCTGATGCAGA   3833
gTaSPL16-B   ------------------------------------------------------------
gTaSPL16-D   ------------------------------------------------------------

gTaSPL16-A   GGCCGGGGAGTCCCCCCTTTTCGAAAAAAAATACATGATTGGAAATATGGAATTGTCTGT   3893
gTaSPL16-B   --------------CCC-------------ATACGTGATTGGAAATATTGAATTGTCTGT   3615
gTaSPL16-D   --------------CCC-------------ATACATGATTGGAAATATGGAATTGTCTGT   4022

gTaSPL16-A   CAGACATATTGTAAATCATGACTCACCTAGCATGTTGACCTATTGCATTGACCTTCTTCG   3953
gTaSPL16-B   CAGACATATTGTAAATCATGACTCACCTAGCATGTTGACCTATCGCATTGACCTTCTTCG   3675
gTaSPL16-D   CAGACATATTGTAAATCATGACTCACCTAGCATGTTGACCTATTGCATTGACCCTCTTCG   4082

gTaSPL16-A   GTACCTTGCAGCTTGTAACCTACCCCGTAGCCTGTTACTTCATGTTGCACATTTGTACCT   4013
gTaSPL16-B   GTACCTTGCAGCTTGTAACCCACCCTGTAGCCTGTTACTTCATGTTGCACATTTTTACCT   3735
gTaSPL16-D   GTACCTTGCAGCTTGTAACCCACCCCGTGGCCTGTTACTTCATGTTGCACATTTG-TACT   4141

gTaSPL16-A   TATTCAGCAATTTTCCTTCTATAAAATAGTCGCACGTGGGATTAGTATTCATAACGAACA   4073
gTaSPL16-B   TATTCAGCAATTTTCCTTCTATAAAATAGTCGCAAGTGGGATTAGTATTCATAACGAACA   3795
gTaSPL16-D   TATTCAGCAACTTTCCTTCTATAAAATAGTCGCAAGTGGGATTAGTATTCATAACGAACA   4201

gTaSPL16-A   AGGATAGCACCTTAGTTTACCCATAAACATACCTAATTAATTGAAGTAATATTCATGGAG   4133
gTaSPL16-B   AGGATAGCACCTTAGTTTACCCATAAACCTACCTAATTAATTAAAGTAATATTTATGGAG   3855
gTaSPL16-D   AGGATAGCGCCTTAGTTTACCCATAAACCTACCTAATTAATTAAAGTAATATTCATGGAG   4261

gTaSPL16-A   GTTGGATGAAGTGTAAATAAATAGAGTAAAAGTTTTACTAGTTCATTAGGAAAAAAGCCA   4193
gTaSPL16-B   GTTGGATGAAGTGTAAATAAATAGAGTAAAAGTTTTACTAGTTCATTAGGAAAAAAGCCA   3915
gTaSPL16-D   GTTGGATGAAGTGTAAATAAATAGAGTAAAAGTTTTACTAGTTCATTAGGAAAAAAGCTA   4321

gTaSPL16-A   CTATTTACCTTGTTCACAATTAGTGCCCTAGTGCTTGAAGCAAGATGCTTCTAGGTTCTG   4253
gTaSPL16-B   CTCTTTACCTTCTTCACAATCAGTGCCCTAGTGCTTGAAGCAAGATGCTTCTAGGTTCTG   3975
gTaSPL16-D   CTCTTTACCT---TCACAATTAGTGCCCTAGTTCTTGAAGCAAGATGCTTCTATGTTCTG   4378

gTaSPL16-A   TAAGATCATATAGATTCCTGCCAAGAGTAAATGTTTAACAAGCTACATGATCATATAGCT   4313
gTaSPL16-B   TAAGATCATATAGATCCCTGCCAAGAGTAAATGTTTAACAAGCTACATGATCATATAGCT   4035
gTaSPL16-D   TAAGATCATATAGATCCTTGCCAAGAGTAAATGTTTAACAAGCTACATGATCATATAGCT   4438

gTaSPL16-A   TG--------------------------------------------TCAAATTTTGAACT   4329
```

```
gTaSPL16-B   TGTTAAATACTACCTCTGTAACTTAACGTAAGACGTTTTTGCAGTGCAAAATTTGAACT   4095
gTaSPL16-D   TGTTAAATACTACCTCTGTAACTTAATGTAAGACG-TTTTTGCAGTTCAAAATTTGAACT   4497

gTaSPL16-A   GCAAAAACGTCTTACATTAAGTAACAGGGGGAGTATCAATTTTCCATAATGCCTGAAGAA   4389
gTaSPL16-B   GCAAAAATGTCTTACATTAAGTAACAGAGGGAGTATCAATTTTCCATAGTGCCTGAAGAA   4155
gTaSPL16-D   GCAAAAATGTCTTACATTAAGTAACAGAGGGAGTATCAATTTTCCATAGTGCCTGAAGAA   4557

gTaSPL16-A   AGAATTTACTCATTCTATCTCCATATGTATCTGTGGCAGTTCCATCATCTTCTTGCCACA   4449
gTaSPL16-B   AGAATTTACTCATTCTATTTCCATATGTATCTGTAGCAGTTCCATTATCTTCTTACCACA   4215
gTaSPL16-D   AGAATTTACTCATTCTATCTCCATATGTATCTGTAGCAGTTCCATTATCTTCTTACCACA   4617

gTaSPL16-A   ACCAATTGACATTGTTGTGAAACCCAGGAACAAGGTTTTCATCATTTCCAACTCCAAGAC   4509
gTaSPL16-B   ACCAATTGACATTGTTGTGAAACCCAGGAACAAGGTTTTCATCATTTCCAACTCCAAGAC   4275
gTaSPL16-D   ACCAATTGACATTGTTGTGAAACCCAGGAACAAGGTTTTCATCATTTCCAACTCCAAGAC   4677
                                        *********************************

gTaSPL16-A   CGGAGCAAAACTGGCAAGGGATCATTAAAACCGAGGAGAACCCCTATTACGCACATCAAC   4569
gTaSPL16-B   CGGAGCAAAACTGGCCAGGGATCATTAAAACTGAGGAGAACCCCTATTACGCACATCAAC   4335
gTaSPL16-D   CGGAGCAAAACTGGCCAGGGATCATTAAAACTGAGGAGAACCCCTATTACGCACATCAAC   4737
             *************** *************** ****************************

gTaSPL16-A   TCCCTCTGGGCATCAGCAGCAGGCAGCATTTTGGTGGATCTGCGTCGACTTACGCCAAAG   4629
gTaSPL16-B   TCCCTCTAGGCATCAGCAACAGGCAGCATTTTGGTGGCTCTGCGTCGACTTACGCCAAAG   4395
gTaSPL16-D   TCCCTCTAGGCATCAGCAACAGGCAGCATTTTGGTCGCTCTGCGTCGACTTACGCCAAAG   4797
             ******* ********** **************** * **********************

gTaSPL16-A   AAGGACGGCGGTTTCCTTTCCTGCAGGAAGGCGAGATAAACTTTGCCACCGGTGTGGCAC   4689
gTaSPL16-B   AAGGACGGCGATTTCCTTTCCTGCAGGAAGGCGAAATAAACTTTGCCACTGGTGTGGCAC   4455
gTaSPL16-D   AAGGACGGCGATTTCCTTTCCTGCAGGAAGGCGAAATAAACTTTGCCACCGGTGTGGCAC   4857
             ********** ********************** *************** **********

gTaSPL16-A   TTGAGCCTTCAGTGTGCCAGCCGCTCCTCAAGACGGTAGCTCCTCCCGAGAGCAGCAGCA   4749
gTaSPL16-B   TTGAGCCTTCAGTGTGCCAACCGCTCCTCAAGACGGTAGCTCCTCCCGAGAGCAGCAGCA   4515
gTaSPL16-D   TTGAGCCTTCAGTGTGCCAGCCGCTCCTCAAGACGGTAGCTCCTCCCGACAGCAGCAGCA   4917
             *******************.***************************** **********

gTaSPL16-A   GCAGCAGCAAGATGTTCTCTGATGGGCTGACTCCGGTGCTCGACTCGGACTGTGCTCTCT   4809
gTaSPL16-B   GCAGCAGCAAGATGTTCTCTGATGGGCTGACTCCAGTGCTGGACTCAGACTGTGCTCTCT   4575
gTaSPL16-D   GCAGCAGCAAGATGTTCTCCGATGGGCTGACTCCGGTGCTCGACTCGGACTGTGCTCTCT   4977
             ******************* **************.***** *****.*************

gTaSPL16-A   CTCTTCTGTCAGCTCCGGCAAACTCCTCCGGTATCGATGTTGGCCAGATGGTCCAGCAGA   4869
gTaSPL16-B   CTCTTCTGTCAGCTCCGGCAAACTCCTCTGGTATCGATGTTGGCCCGATGGTCCAACAGA   4635
gTaSPL16-D   CTCTTCTGTCAGCTCCGGCAAACTCCTCCGGTATCGATGTTGGCCAGATGGTCCAACAGA   5037
             **************************** ****************.*********.****

gTaSPL16-A   CTGAACACATCCCCATTGCCCAGCCTCTGTTCTCCAACCTGCAGTTCAGCAGCTCGTCCT   4929
gTaSPL16-B   CTGAACACATCCCCATTGCCCAGCCTCTGTTCTCCAACCTGCAATTCAGCAGCTCGTCCT   4695
gTaSPL16-D   CTGAACACATCCCCATTGCCCAGCCTCTGTTCTCCAACCTGCAGTTCAGCAGCTCGTCCT   5097
             *******************************************.****************

gTaSPL16-A   GGTTCTCGCGCACCCAGGCTTCCACCGGCACCGTCTCAGCGACCGGATTTTCCTGCCCCG   4989
gTaSPL16-B   GGTTCTCGCGCACCCAGGCTTCCACCGGTACCGTCTCAGCGACTGGATTTTCCTGCCCTG   4755
gTaSPL16-D   GGTTCTCGCGCACCCAGGCTTCCACCGGCACCGTCTCAGCGACCGGATTTTCCTGCCCCG   5157
             *******************************************.****************
```

```
gTaSPL16-A   CGGTGGAGAATGAGCAACTGAACAATGTGCTAAGCTCGGACAACAATGACTTGAACTACA   5049
gTaSPL16-B   TGGGGGAAAATGAGCAACTGAACAATGTGCTAAGCTCGGACAACAATGACTTGAACTACA   4815
gTaSPL16-D   CGGTGGAAAATGAGCAACTGAACAATGTGCTAAGCTCGGACAACAATGACTTGAACTACA   5217
              ** ***.****************************************************

gTaSPL16-A   ATGGGATATTTCATGTCGGCGGTGAAGGCTCGTCGGACGGCGCCCCGCCGTCTCTGCCCT   5109
gTaSPL16-B   ATGGGATATTTCATGTCGGCGGTGAAGGCTCGTCGGACGGCGCCCCGCCATCTCTGCCCT   4875
gTaSPL16-D   ATGGGATATTTCATGTCGGCGGTGAAGGCTCCTCGGACGGCGCCCCGCCATCTCTGCCCT   5277
             ******************************* *****************.**********

gTaSPL16-A   TCCCGTGGCAGTAGTTTTCAGTGGC                                      5134
gTaSPL16-B   TCCCGTGGCAGTAGTTTTCAGTGGC                                      4900
gTaSPL16-D   TCCCGTGGCAGTAGTTTTCAGTGGC                                      5302
             *************************
```

Figure S4. Genomic DNA Sequences Alignment. *gTaSPL16-A*, *gTaSPL16-B* and *gTaSPL16-D* represent the genomic DNA sequences of *TaSPL16* homoeolog from A, B and D subgenome. Sequences were aligned by ClustalW. Exon sequences were highlighted in yellow and the consensus were shown by astertisks. Start codons and stop codons were indicated in red letters. miR156 target sites were highlighted in pink.

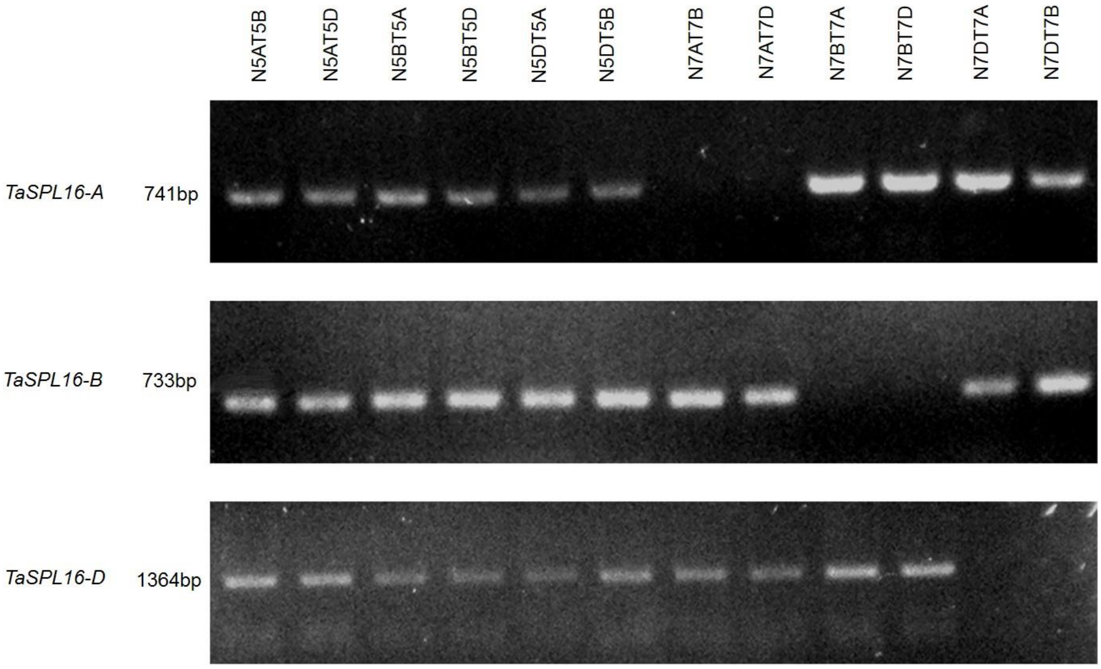

Figure S5. Chromosome localization of the three *TaSPL16* homoeologs using Chinese Spring nullisomic-tetrasomic (NT) lines and genome-specific primer pairs. Chinese Spring NT lines, a series of lines with each missing one pair of chromosomes that is replaced by an extra pair of homoeologous chromosomes, for an example, N5AT5B indicates the line with missing one pair of 5A chromosomes that is replaced by an extra pair of 5B chromosomes. The names of the NT lines used were given above individual lanes. *TaSPL16-7A*, *TaSPL16-7B*, and *TaSPL16-7D* in left refer to the corresponding subgenome-specific primer pair used to perform PCR, the PCR products for each primer pair/NT line are shown in right.

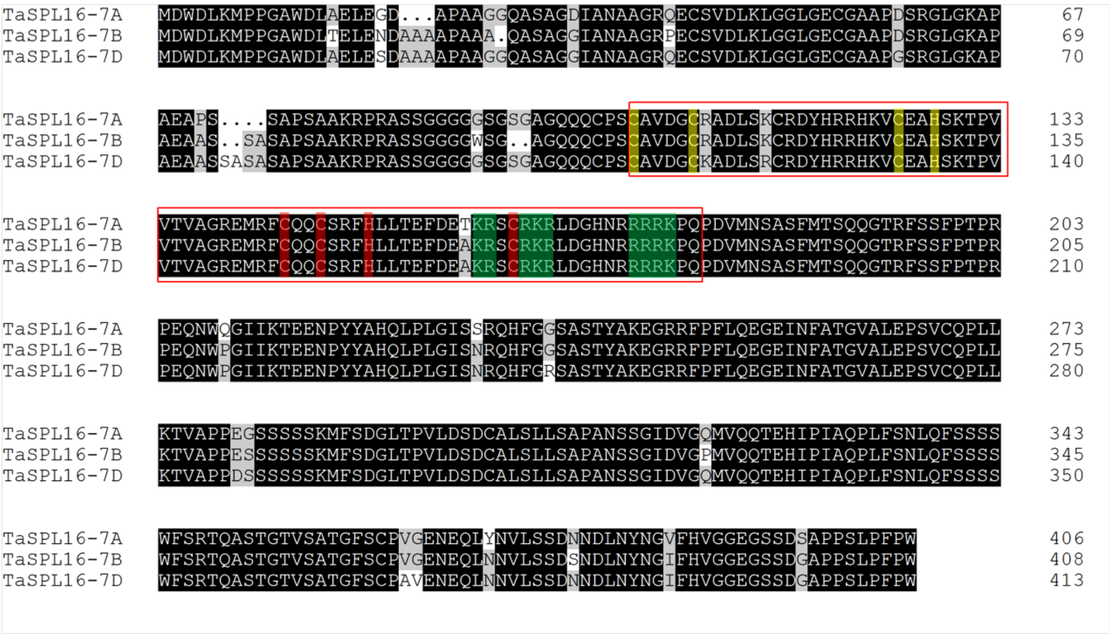

Figure S6. Sequence alignment of wheat TaSPL16-7A, TaSPL16-7B and TaSPL16-7D produced by ClustalW. SBP domain was indicated in red box, and the two zinc finger motifs (Zn-1 and Zn-2) and nuclear localization signal (NLS) were marked by yellow, red and green boxes, respectively.

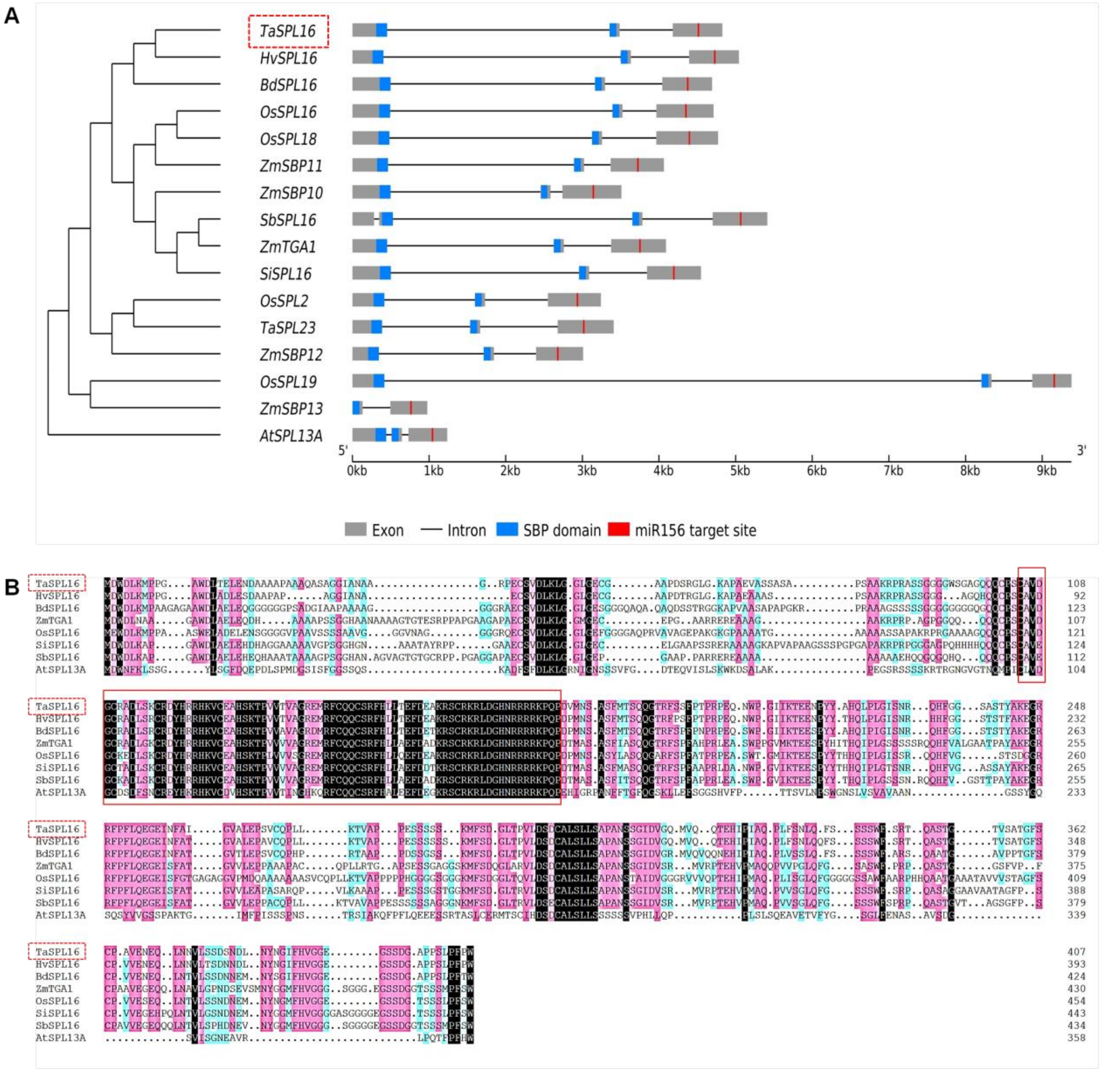

Figure S7. Structural characterization of SPL16 genes and amino acid alignment of TaSPL16 and its orthologous genes. (A) Structural characterization of the *SPL16* genes and the other homologous genes that belong to the same group as *TaSPL16* in the phylogenetic tree shown in Figure 1. Black boxes indicated exons while introns was indicated with black lines. The SBP domain was shown with blue boxes, and the miR156 binding site with red boxes. (B) Amino acid alignment of TaSPL16 and its orthologous proteins. The numbers on the right indicated the position of the residues in the full protein. Dark boxes represent identical residues, pink boxes indicate conserved residues, and variant residues are indicated by blue boxes or none. The red box presents the *Squamosa* promoter-binding (SBP) domain of these SPL proteins.

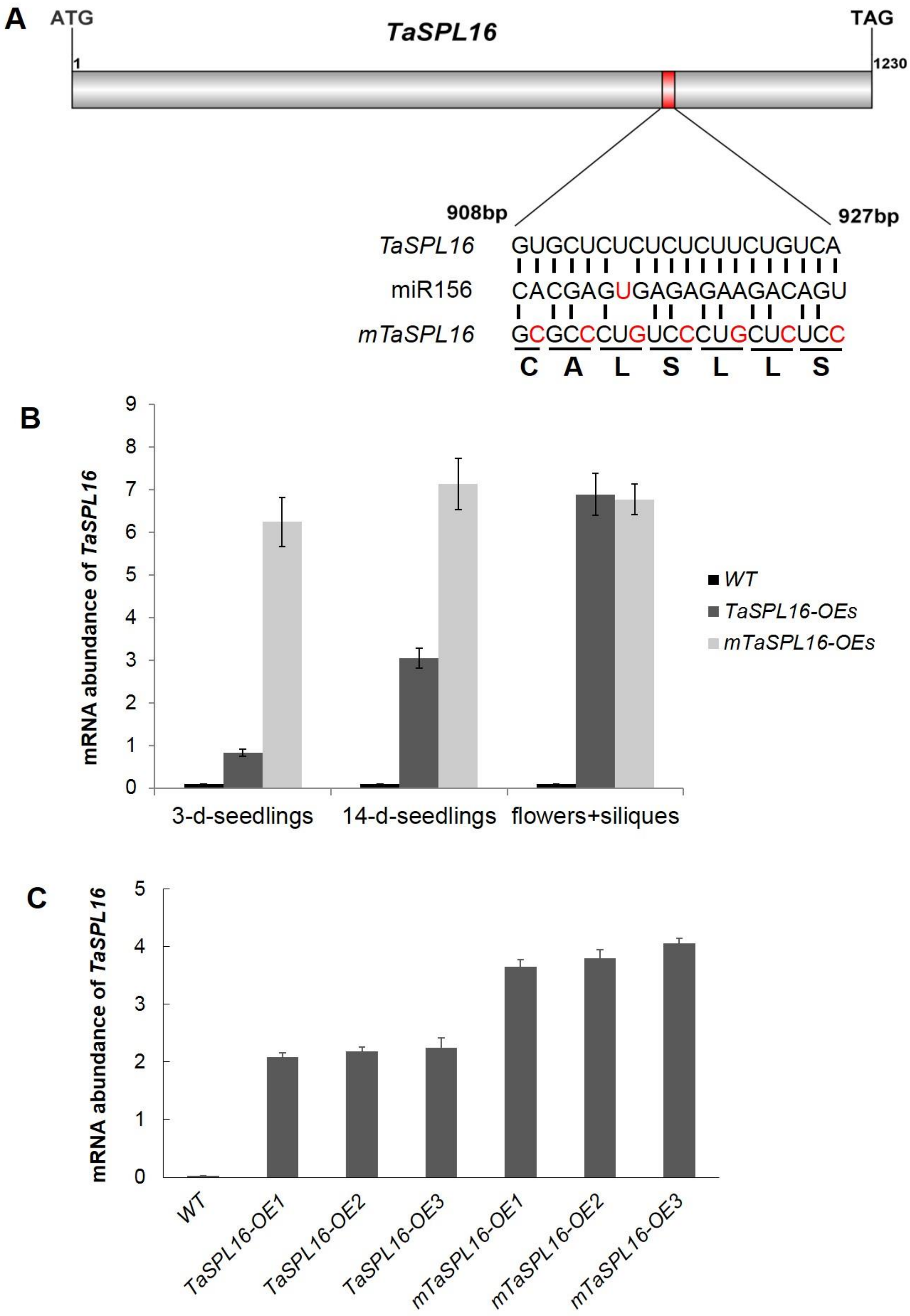

Figure S8. The mRNA abundance of *TaSPL16* in transgenic *Arabidopsis* lines. (A) The sequence of *TaSPL16* and *mTaSPL16* at miR156 binding site. (B) The mRNA abundance of *TaSPL16* in wild type plants and transgenic lines *TaSPL16-OEs and mTaSPL16-OEs*. The values of individual columns were averages of five independent homozygous transgenic lines, bars represent standard deviation. (C) Relative mRNA abundance of *TaSPL16* in each of three independent transgenic *TaSPL16-OE* lines and each of three independent transgenic *mTaSPL16-OE* lines at 14-day seedlings detected by quantitative real-time reverse transcriptase-polymerase chain reaction, *Tubulin beta 2* (AT5G62690) being used for normalization.

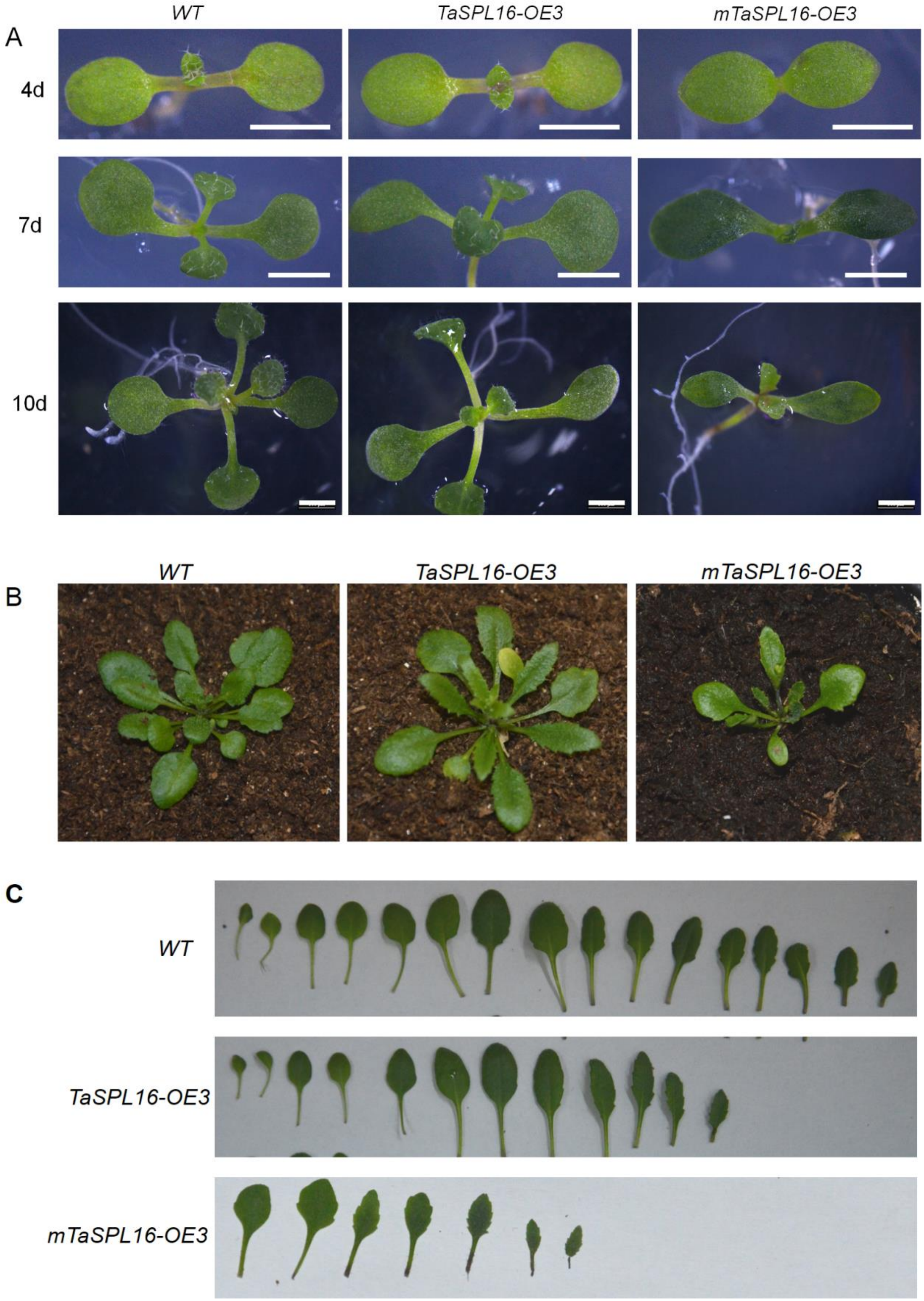

Figure S9. Phenotypes of wild type plants and homozygous transgenic lines of *Arabidopsis* at post-germination and seedling stages. WT, wild type; *TaSPL16-OE3*, a transgenic line expressing non-mutated *TaSPL16-7B*; *mTaSPL16-OE3*, a transgenic line expressing miR156-resistance mRNA of *mTaSPL16-7B*. *TaSPL16-OE3* and *mTaSPL16-OE3* are shown as representatives because similar phenotypes were observed among independent *TaSPL16-OE* lines or independent *mTaSPL16-OE* lines. (A) The phenotypes of wild type and transgenic seedlings at 4-day, 7-day and 10-day post-germination. Compared to wild type and *TaSPL16-OE3*, *mTaSPL16-OE3* seedlings exhibited a delay in the emergence of vegetative leaves (3-4 days late).

Bars represent 2mm. (B)-(C) The phenotypes of wild type and transgenic seedlings and leaves at 16 days post-germination. *mTaSPL16-OE3* and *TaSPL16-OE3* seedlings showed a significant decrease in leaf initiation rate, compared to wild type.

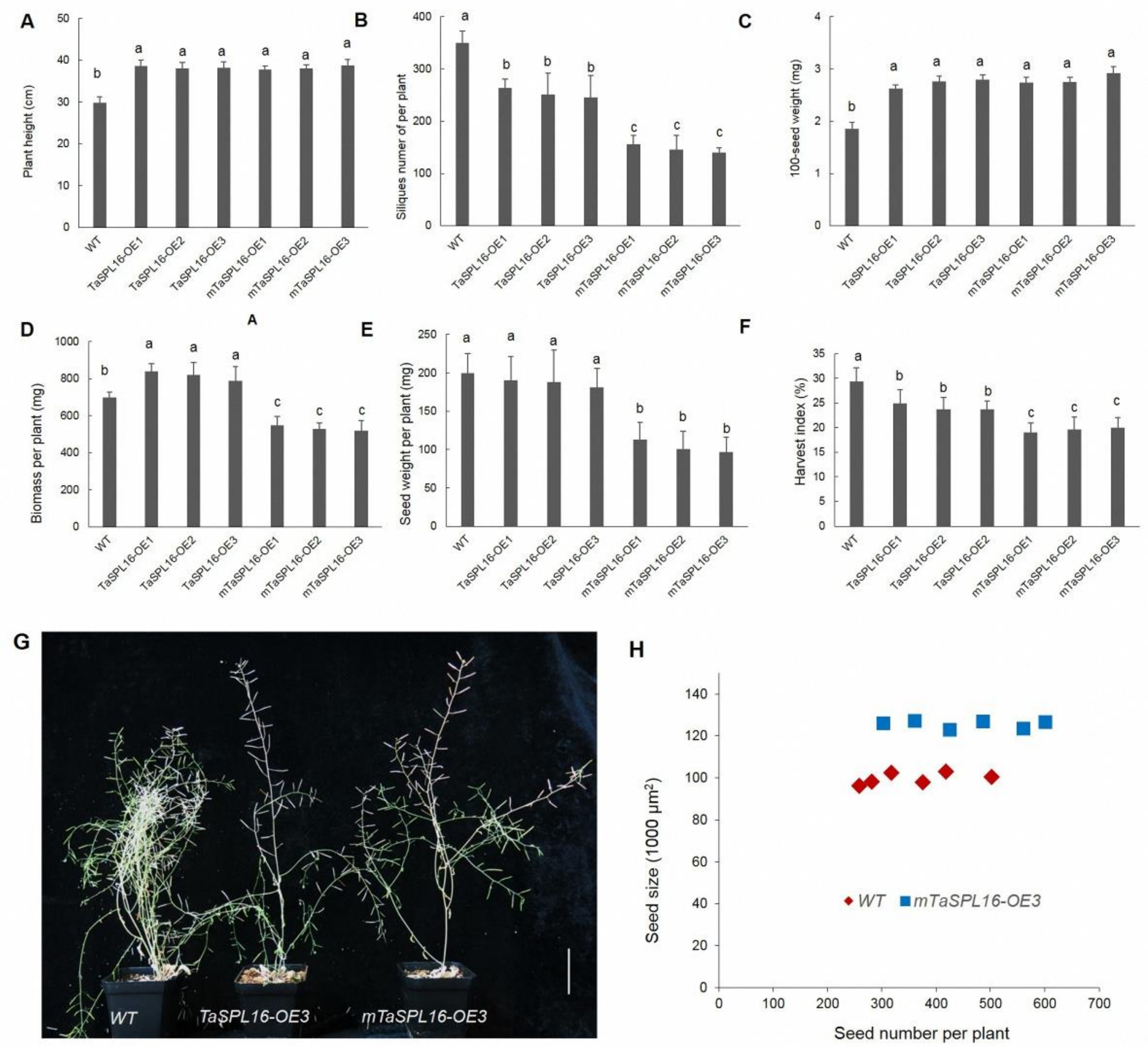

Figure S10. Ectopic expression of *TaSPL16* in *Arabidopsis* influences yield-related traits. A-F, Comparisons of plant height, total silique number per plant, 100-seed weight, seed weight per plant, Biomass per plant, and harvest index, respectively. WT, wild type *Arabidopsis*; *TaSPL16-OEs*, transgenic lines expressing non-mutated *TaSPL16-7B*; *mTaSPL16-OEs*, transgenic lines expressing miR156-resistance mRNA of *mTaSPL16-7B*. The values of each column represent means ± SD (n=10). Different letters at top of each column indicate a significant difference among genotypes at P<0.05 determined by Tukey's HSD test. G, The photos of plant at maturity. bar indicate 5cm. H, seed size (represented by the projective areas of a seed) between wild type and *mTaSPL16-OE3* transgenic line which only 5-10 siliques were allowed to develop on the main inflorescence, resulting in 259-506 (WT) or 305-600 seeds (*mTaSPL16-OE3*) obtained per plant. Each point represents the average value of one seed from one plant. Values represent means ± SE (n=10).
